# Nanoscale electrodeposition: Dimension control and 3D conformality

**DOI:** 10.1002/EXP.20210012

**Published:** 2021-11-07

**Authors:** Sol A Lee, Jin Wook Yang, Sungkyun Choi, Ho Won Jang

**Affiliations:** ^1^ Department of Materials Science and Engineering, Research Institute of Advanced Materials Seoul National University Seoul 08826 Republic of Korea; ^2^ Advanced Institute of Convergence Technology Seoul National University Suwon 16229 Republic of Korea

**Keywords:** 3D conformality, dimension control, electrochemistry, electrodeposition, energy conversion devices, nanostructures

## Abstract

Electrodeposition with a long history has been considered one of the important synthesis techniques for applying various applications. It is a feasible route for fabricating nanostructures using diverse materials due to its simplicity, cost‐effectiveness, flexibility, and ease of reaction control. Herein, we mainly focus on the nanoscale electrodeposition with respect to dimension control and three‐dimensional (3D) conformality. The principles of electrodeposition, dimensional design of materials, and uniform coatings on various substrates are presented. We introduce that manipulating synthesis parameters such as precursors, applied current/voltage, and additives affect the synthesis reaction, resulting in not only dimensional control of materials from three‐dimensional structures to zero‐dimensional atomic‐level but also conformal coatings on complicated substrates. Various cases regarding morphology control of metal (hydro)oxides, metals, and metal–organic frameworks according to electrodeposition conditions are summarized. Lastly, recent studies of applications such as batteries, photoelectrodes, and electrocatalysts using electrodeposited materials are summarized. This review represents significant advances in the nanoscale design of materials through methodological approaches, which are highly attractive from both academic and commercial aspects.

## Graphical abstract

This review mainly focuses on the nanoscale electrodeposition with respect to dimension control and three‐dimensional (3D) conformality. The overall principles of electrodeposition are presented, and the feasible dimensional/morphological control of various materials and the uniform coatings on various substrates are introduced.

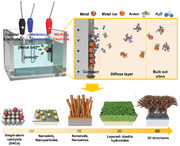

## 1 INTRODUCTION

Along with the technological revolution, nanotechnology, which is a technology conducted at a nanoscale, has been applied to contemporary a lot of industries (e.g., electronics, energy, medicine, environment, food, textile). Precise control of tiny systems developed the understanding of distinctive physicochemical properties of materials compared to their intrinsic properties from bulk. Various synthetic approaches such as physical/chemical deposition methods (e.g., chemical vapor deposition (CVD), atomic layer deposition, e‐beam deposition) and solution processes are being used in conjunction with nanotechnology. Among them, electrodeposition is a well‐established technology with a very long history. Predating 2000 years, it is hypothesized that electroless deposition was conducted to form gold coatings on a copper surface using the different activity of metals.^[^ ^1^ ^]^ In 1800, Alessandro Volta first demonstrated the continuous current flow in an electrochemical cell by converting electrolytic chemical sources into electricity.^[^ ^2^ ^]^ Humphry Davy first discovered the alkali metals such as potassium and sodium by passing an electric current through molten salts.^[^ ^3^ ^]^ In 1946, Grace Riddell and Abner Brenner discovered that a chemical reducing agent in nickel electroplating baths provides electrons for the electrochemical reaction without external current, called electroless plating.^[^ ^4^ ^]^

With the rapid growth of electrodeposition methods and efforts to uncover the reaction mechanisms, electrodeposition has become a promising synthesis technology to be adjusted for diverse applications for the following advantages. First, a lot of materials can be synthesized under ambient conditions compared to the existing physical/chemical synthesis methods. For example, CVD is conducted under high temperatures, and sputter or e‐beam deposition requires high vacuum states, which consumes huge electricity, even not synthesizing materials. Second, uniform films having a variety of morphologies can be synthesized through electrodeposition. By taking advantage of electrodeposition, it is possible to form conformal coatings on substrates having complicated three‐dimensional (3D) nanostructures. Third, it is easy to control the composition of the electrodeposits using various types of precursors in the electrolytes, and reactions can be controlled in real‐time. Fourth, electrodeposition is cost‐effective synthesis technology and facilitates scale‐up, which is appropriate for the industrial process.^[^ ^5^ ^]^ However, electrodeposition is not applicable to insulating substrates. It also possesses environmental concerns by using and disposing of toxic plating solutions and having a high risk of hydrogen embrittlement.

In this review article, we demonstrate the nanoscale electrodeposition for controlling dimensions and conformal coating. We introduce fundamental principles of nanoscale electrodeposition and distinctive morphologies of various materials. Lastly, energy‐conversion applications using electrodeposition are introduced.

## 2 ELECTRODEPOSITION

### 2.1 Principles of electrodeposition

**FIGURE 1:**
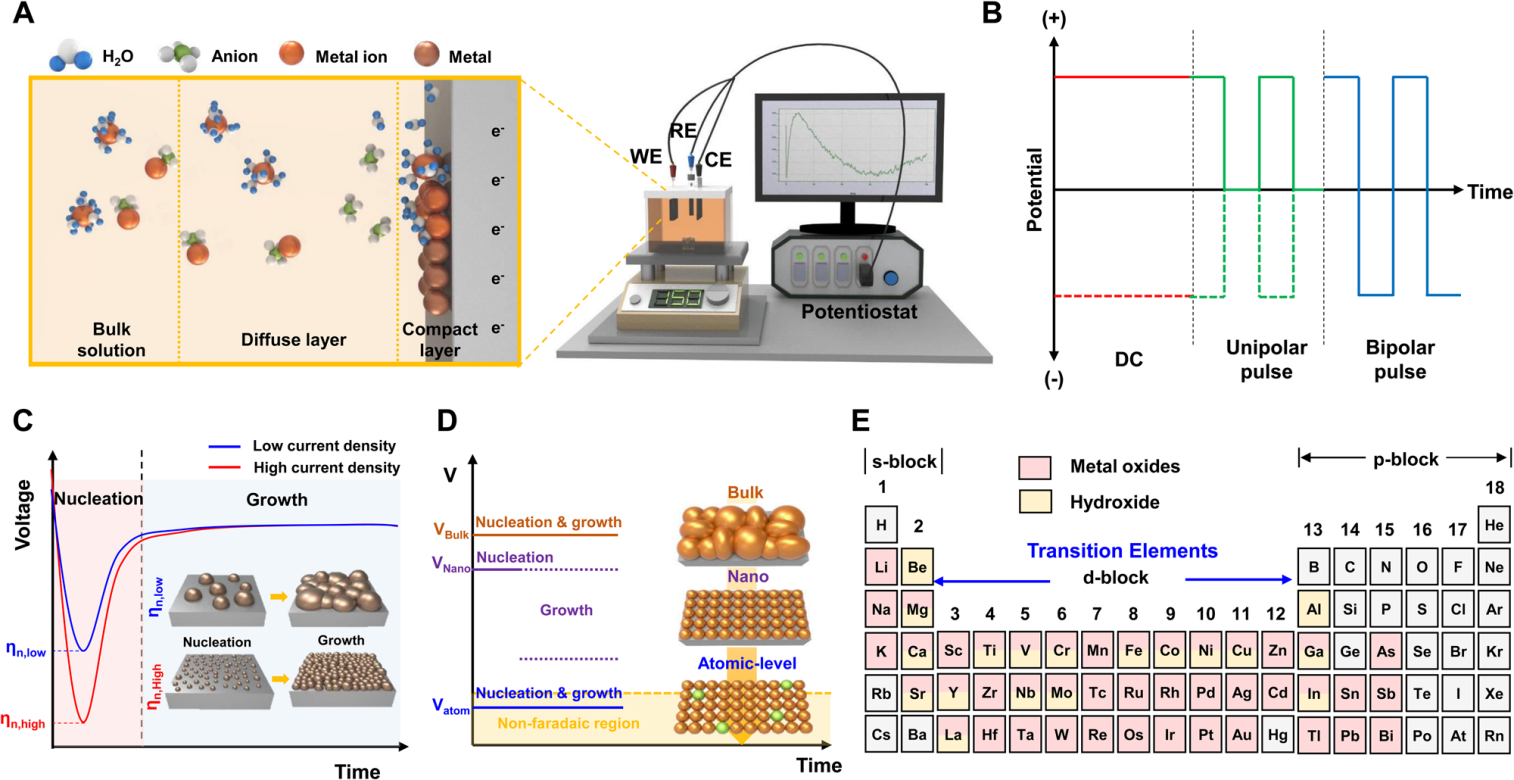
(A) Schematic of electrodeposition and electrical double layer (EDL). (B) Modes of electrodeposition. (C) Voltage‐time transient curves of electrodeposition and schematic illustrating the nucleation and growth of films by varying deposition overpotentials. (D) Comparison of bulk to atomic‐level electrodeposition. (E) Possible electrodeposited products. Adapted with permission.^[10]^ Copyright 2020, Royal Society of Chemistry.

Electrodeposition can be conducted under the two‐electrode system or three‐electrode system. Two‐electrode configuration consisting of an anode and cathode is simple, but the current or voltage is changing during the reaction because of the charging of the electrode surface. Therefore, the three‐electrode system is come up with a solution for applying exact current/potential to the electrode during the operation. Figure 1A shows the three‐electrode electrochemical synthesis system consists of the working electrode (WE), counter electrode (CE), reference electrode (RE), and electrolytes. A RE having a stable and specific electrode potential is employed to apply an accurate potential to the WE.

Before conducting electrodeposition, the electrolytes are prepared by dissolving reactants to be deposited. The plausible parameters in the chemical bath for affecting the morphology of electrodeposits are temperature, concentration of the reactants, pH, additives, and so on. Electrodeposition occurs near the electrode surface, especially the nanoscale electric double layer (EDL) with the potential gradient of 10^5^ V cm^–1^.^[^ ^6^ ^]^ Figure 1A shows the nucleation and growth of films in aqueous electrolytes. Near the electrode surface, a diffusion layer called the mass transport boundary layer is formed. Its thickness can vary depending on the viscosity of the solution and the stirring rate. The transport of the species toward the electrode surface is controlled by diffusion. The metal species are reversibly interconverted in the diffusion layer and bulk electrolyte. Therefore, precursor species lie in dynamic equilibrium conditions by hydrated complexes. As a result, an EDL is formed at the electrode surface. The adsorbed water molecules and positively charged species are attached to the electrode surface. In the EDL, metal complexes can react by electron transfer and discharge at the electrode surface. Finally, nucleation occurs on the electrode surface, and formed nuclei grow to deposit films.

As shown in Figure 1B, the synthesis process can be differed by adjusting various electrodeposition modes. A simple mode uses direct current/potential, where the parameters are applied current/potential and deposition time. In the case of DC electrodeposition, after the nuclei are formed, the reaction proceeds to promote growth on the existing electrodeposits. During electrodeposition, the consumed ions cause a concentration gradient and affect the roughness of the films. The other way is applying pulse mode, and pulse electrodeposition can be classified as unipolar, where all pulses are in the same direction, and bipolar with both anodic and cathodic pulse. In pulse electrodeposition, the plausible parameters are on‐time (*T*_on_), off‐time (*T*_off_), pulse current density, and time. The ratio of the *T*_on_ to the sum of *T*_on_ + *T*_off_ is called the duty cycle. Compared to the continuous electrodeposition, more uniform films can be deposited since the ions can be replenished during the off‐time, leading to the decreased concentration gradient.^[^ ^7^ ^]^ This tendency can be more pronounced in bipolar electrodeposition, in which ions are continuously supplemented, than in unipolar electrodeposition, where ions can be replenished during *T*_off_. Also, the pulse mode promotes new nucleation to make fine electrodeposits. By taking advantage of pulse electrodeposition, uniformity in areas such as edges with more electricity can be improved. As selected research, Yang et al. compared the DC electrodeposition and pulse plating on lithium electrodeposition.^[^ ^8^ ^]^ They showed the effects of controlling the current density of DC electrodeposition and duty cycle for pulse electrodeposition on the shape of the lithium films. When DC current increases from 0.1 to 10 mA cm^–2^, the morphology of lithium particles changed from rock‐like to fiber‐like. In the case of pulse electrodeposition (*T*_on _= 0.4 ms, *T*_off _= 5 ms), electrodeposited films with pulse showed a thicker needle‐like structure compared to the DC electrodeposits. Su et al. demonstrated the nanocrystalline cobalt electrodeposits with different morphologies via four electrodeposition modes of DC, unipolar pulse, reverse pulse, and bipolar pulse electrodeposition.^[^ ^9^ ^]^ Among four types of electrodeposition, conformal coating surfaces with the fine granular structure were achieved using bipolar pulse electrodeposition.

The dissolved reactants can be oxidized or reduced by controlling the applied cell potential or deposition current, continuously deposited to the WE surface in real‐time.^[^ ^10^ ^]^ In the case of tuning the current for the electrodeposition process, the nucleation rate of the crystal can be controlled, resulting in electrodeposits with good adhesion, controlled morphology, and amount of the electrodeposits. The charges consumed for the electrodeposition can be calculated by the multiple of time and current. When applying the potential, a rapid decrease of cell current can be observed because of the diffusion‐limited reactants from the bulk electrolyte to the electrode surface.

The crystal growth rate can be controlled by changing the deposition overpotential, which is defined as the difference between the applied deposition potential and the potential regarding crystal growth.^[^ ^11^ ^]^ The control of growth overpotential affects the nucleation and growth of the films, resulting in different film morphology (Figure 1C). As selected reports, Yi Cui group demonstrated the lithium nucleation on Cu and growth by varying the electrochemical overpotentials.^[^ ^12^ ^]^ The critical nuclei radius is inversely proportional to the electrochemical overpotential of Li, and areal nuclei density is proportional to overpotential cubed. The deposited Li nuclei showed a decreasing size and were more closely packed as the cathodic current density increases from 0.1 to 10 mA cm^–2^. Similar behavior was observed by Park et al., where surface morphology of Ni(OH)_2_ films was dependent on the deposition potentials.^[^ ^13^ ^]^ The higher deposition overpotentials can be regarded as the driving force for the heterogeneous nucleation process, resulting in a dense and thin layer. In the deposition of Ni(OH)_2_ at a low current density, the deposition potentials were consumed for the charging current or non‐faradaic currents. Therefore, less nucleation of Ni(OH)_2_ and growth of Ni(OH)_2_ occurred on the surface of nuclei and resulted in porous surface morphology.

By controlling the nucleation and growth processes, it is possible to obtain the electrodeposits from bulk to atomic scale. In typical electrodeposition, the redox reactions of materials occur in the area beyond non‐faradaic currents, which are required to fill the electrical double layer. As shown in Figure 1D, where nucleation and growth processes are conducted higher than the non‐faradaic region, bulk to nanoscale electrodeposits can be formed. Nanoscale electrodeposits can be obtained by controlling the nucleation/growth time and potential. If electrodeposition is performed in the underpotential region, atomic‐scale electrodeposition can occur.

One of the most advantageous features of electrodeposition is that a lot of materials can be electrodeposited having various phases. Figure 1E shows the possible form of electrodeposits according to the periodic table. Various metal oxide and hydroxides can be obtained from a lot of elements by adjusting the deposition conditions. Furthermore, it is possible to deposit alloys by dissolving the reagents in the electrolyte under an appropriate potential window. Table 1 shows the standard electrode potential, which is commonly written as a standard reduction potential, of various metals versus standard hydrogen electrode (SHE).^[^ ^14^ ^]^ A standard electrode potential E° is defined as the potential of a half‐reaction relative to a RE at a temperature of 25°C and a pressure of 1 atm. In the case of solvent water, the common RE is SHE, where the E° is assigned to 0 V at all temperatures for the half‐reaction as follow:

(1) $$2H^{+}\left(\mathrm{aq}\right)+2e^{-}\to H_{2}\left(g\right)$$

**TABLE 1:** Summarized reduction potentials of transition metals.^[^ ^14^ ^]^

**Metal**	**Reaction**	**E° (V vs. SHE)**
Mn	Mn^2+^ + 2e^–^ → Mn	−1.18
Zn	Zn^2+^ + 2e^–^ → Zn	−0.76
Fe	Fe^2+^ + 2e^–^ → Fe	−0.44
Co	Co^2+^ + 2e^–^ → Co	−0.28
Ni	Ni^2+^ + 2e^–^ → Ni	−0.24
Bi	Bi^3+^ + 3e^–^ → Bi	+0.31
Cu	Cu^2+^ + 2e^–^ → Cu	+0.34
Ru	Ru^2+^ + 2e^–^ → Ru	+0.6
Rh	Rh^3+^ + 3e^–^ → Rh	+0.76
Ag	Ag^+^ + e^–^ → Ag	+0.80
Pd	Pd^2+^ + 2e^–^ → Pd	+0.92
Ir	Ir^3+^ + 3e^–^ → Ir	+1.0
Pt	Pt^2+^ + 2e^–^ → Pt	+1.18
Au	Au^3+^ + 3e^–^ → Au	+1.50

As the positive value of reduction potential becomes larger, the element is easily reduced, indicating a good oxidizing agent. The larger negative reduction potential of the element tends not to be easily reduced. By using different reduction potentials of materials, it is possible to fabricate galvanic cell, which generates the electricity by a spontaneous redox reaction.

Furthermore, it is possible to assume the proper potential ranges to be applied for multi‐component electrodeposition. If the reaction potential of the element shows a significant difference, we have to enlarge the potential window by choosing a solvent having a wide potential range, using pulse mode, or adding additives.

### 2.2 Morphology control

**FIGURE 2:**
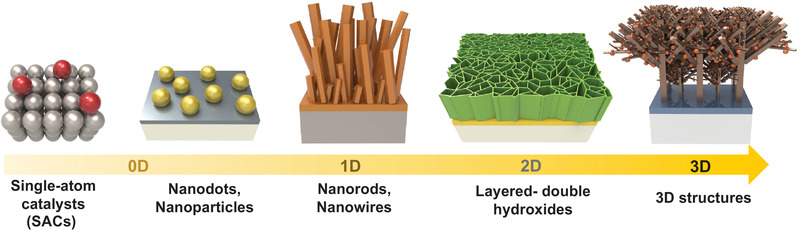
Schematic illustrating the dimension‐controlled electrodeposits

The possible morphologies of electrodeposits are limitless, from complicated three‐dimensional (3D) nanostructures to single‐atom (0D), as shown in Figure 2. The dimension and morphologies of the materials can be tuned by controlling various factors such as concentration, kinds of additives, voltage/currents, and so on (Table 2). Hierarchical 3D nanostructures such as flower‐like structures, nanoporous films can offer an extremely high surface area with an interconnected network.^[^ ^15^ ^]^ For example, various dendrites can be synthesized via electrodeposition methods, and they are applicable for energy conversion devices, catalysis, and so on. Various elements such as Cu,^[^ ^16^ ^]^ Au,^[^ ^17^ ^]^ Co,^[^ ^18^ ^]^ Zn,^[^ ^19^, ^20^ ^]^ Bi,^[^ ^21^ ^]^ Ni,^[^ ^22^ ^]^ etc., can be electrodeposited to form hierarchical structures by limited growth under non‐equilibrium conditions. One of the plausible methods for hierarchical nanostructures is hydrogen bubble dynamic template deposition (HBDT) without using additional templates. At a relatively large cathodic potential, both metal cations and the protons are reduced to generate hydrogen bubbles which act as a dynamic template during the electrodeposition.^[^ ^15^, ^23^ ^]^ As selected studies using HBDT for metallic nanostructural films, Kang's group synthesized 3D open porous copper films where the morphology of the films can be tuned by applied current density/voltage.^[^ ^24^ ^]^ Zhuo et al. synthesized highly porous Ni‐Sn alloy films by varying the Sn precursor concentration.^[^ ^25^ ^]^ By applying cathodic overpotential of −4 V, reduction of Ni and Sn ions and hydrogen evolution reaction was accompanied simultaneously, leading to the 3D porous structures with a large surface area.

**TABLE 2:** Summarized materials with controlled morphologies

**Dimension**	**Materials**	**Morphology**	**Parameter**	**Ref**.
3D	Cu	Porous film	Applied potentials	^[^ ^24^ ^]^
	Au	Dendrite	Additive	^[^ ^17^ ^]^
	Co	Dendrite	Applied potential	^[^ ^18^ ^]^
	Zn	Dendritic leaf	Overpotentials	^[^ ^16^ ^]^
2D	Ni(OH)_2_	Nanosheets	Applied current	^[^ ^13^ ^]^
	NiFe LDHs	Nanosheets	Applied current	^[^ ^29^ ^]^
	BiOI	Nanosheets	Concentration, pH	^[^ ^50^ ^]^
1D	ZnO	Nanorods/nanowires	Concentration	^[^ ^191^ ^]^
	In(OH)_3_	Nanorods	Additives	^[^ ^192^ ^]^
0D	Ni	Nanoparticles	Additives	^[^ ^27^ ^]^
	Cu_2_O	Nanocrystals	Concentration	^[^ ^16^ ^]^
	Pt	Single atom	Applied potential	^[^ ^38^ ^]^

Two‐dimensional (2D) metal hydroxides and metal nanoplates, which are hard to be synthesized using physical/CVD techniques, can be electrodeposited with flexible tunability of metal ions. Compared to the direct electrodeposition of metals and metal oxides, metal hydroxides undergo a two‐step electrochemical reaction, according to the following equations.^[^ ^10^, ^26^ ^]^

(2) $${\mathrm{ClO}}_{3}^{-}+3H_{2}O+6e^{-}\to {\mathrm{Cl}}^{-}+6{\mathrm{OH}}^{-}$$

(3) $${\mathrm{NO}}_{3}^{-}+H_{2}O+2e^{-}\to {\mathrm{NO}}_{2}^{-}+2{\mathrm{OH}}^{-}$$

(4) $${\mathrm{IO}}_{3}^{-}+3H_{2}O+6e^{-}\to I^{-}+6{\mathrm{OH}}^{-}$$

First, anions are electrochemically reduced in the presence of water to produce hydroxide ions near the cathode surface. Then metal cations in the bath solution are simultaneously combined with OH^−^ at the WE surface as follow:

(5) $$M^{x+}+{\mathrm{xOH}}^{-}\to M{\left(\mathrm{OH}\right)}_{x}$$

With the cathodic electrodeposition, not only single metal hydroxides but also multi‐component metal hydroxides such as NiFe‐, NiCo‐, and NiCoFe‐hydroxides can be successfully achieved.^[^ ^26^, ^27^, ^28^, ^29^, ^30^ ^]^

One‐dimensional (1D) nanorods or nanowires having an anisotropic structure can be obtained via electrochemical deposition technique. The coverage, diameter, and height of the nanorods/nanowires can be controlled by changing the precursor concentration, bath temperature, additives, and so on. The representative ZnO nanorods/nanowires having a hexagonal structure can be conformally formed on the conductive substrates.^[^ ^31^ ^]^ Penner's group discovered the electrochemical step‐edge decoration (ESED) to obtain metal nanowire arrays on the basal plane of highly oriented pyrolytic graphite (HOPG) electrodes.^[^ ^32^ ^]^ As a representative work, they synthesized molybdenum (Mo) nanowires having a diameter from 15 nm to 1 μm using ESED.^[^ ^33^ ^]^ As‐deposited molybdenum oxides (MoO*_x_*) were reduced in hydrogen and become metallic Mo nanowires.

Furthermore, nanoparticles or nanodots (0D) with controlled properties such as size and morphology can be produced by electrodeposition.^[^ ^34^ ^]^ In comparison to other synthesis methods, nanoparticles are directly attached to the substrate, and morphology and crystallographic orientations can be easily tuned by adjusting the chemical baths and operating conditions such as pH, temperature, additives, and so on. The size of nanoparticles or nanocrystallites can be tuned by controlling the nucleation and growth procedure. Crystal shape or orientation is determined toward minimum overall surface free energy at equilibrium condition.^[^ ^35^ ^]^ Crystal growth direction is familiar to the lower energy surfaces, resulting in reduction/elimination of higher surface energy by growing faster perpendicularly to the high energy plane. In fact, actual crystal shapes often show discrepancy to the predicted shape because of the interaction of the species in the growth environments, such as impurities and ions, which affect the growth rate of crystals thermodynamically and kinetically. The preferential adsorption of species affects the growth rate of an anisotropic crystal plane, leading to the crystal shape. Choi's group demonstrated the rational control of cuprous oxide and zinc metal shape via the electrodeposition method.^[^ ^16^ ^]^ They controlled the crystal shape by tuning the crystal habit and branching growth limited by mass transport. The modification of crystal shape was exploited by introducing proper additives, resulting in preferential adsorption to adjust the crystal habit. By controlling the specific crystallographic planes, Cu_2_O crystals were synthesized with various crystal shapes from octahedral to cubic. Under non‐equilibrium conditions, crystal growth can be promoted by controlling branching growth. If the crystal growth is so fast that the ions are depleted, and a depletion zone is formed, the crystal growth is limited by mass transport. By increasing the overpotential, dendritic zinc and Cu_2_O crystals were obtained. At a very low deposition overpotential, polyhedral crystals with void space were obtained.

The electrodeposition process can form the electrodeposits from the nanoscale to atomic level using surface‐limited techniques called underpotential deposition (UPD). The single‐atom layer and single‐atoms (0D) could be deposited on a foreign substrate at potentials positive with regard to equilibrium potential.^[^ ^36^, ^37^, ^38^ ^]^ The bindings of the monolayer to the foreign substrate are stronger than monolayer‐monolayer bindings, causing the self‐limiting behavior of UPD. The single atoms can be site‐specifically deposited on a supporting substrate by forming energetically favorable metal–support bonds with self‐termination.

### 2.3 3D conformality

**FIGURE 3:**
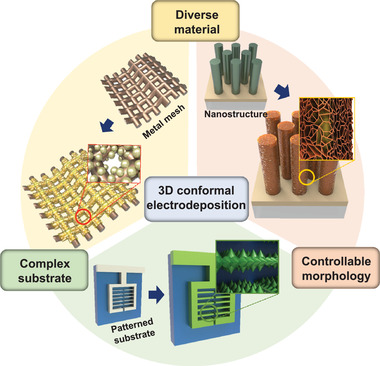
Schematic of 3D conformal coatings via electrodeposition

One of the powerful advantages of electrochemical deposition methods is that they can form conformal coatings to complicated 3D structures (Figure 3). Since the ions in the electrolyte are directly attached to the substrate by electric power, uniform films can be formed on the substrates having a large surface area. Coating porous substrates with a large surface‐to‐volume ratio with different materials have become a promising route for obtaining functionalized nanomaterials. The kinds of representative 3D substrates having complex structural design are conductive and microporous substrates (e.g., metal foam, woven carbon cloth, metal meshes), synthesized nanostructures (e.g., nanorods, nanowires, pyramids, nanopillars, nanotubes), and patterned substrates. For the selected examples of electrodeposition on the conductive and microporous substrate, Li et al. electrochemically synthesized Fe‐containing CoFe, NiFe, and LiFe layered double hydroxide (LDH) array on Ni foams.^[^ ^39^ ^]^ In the case of NiFe‐LDH, showing the highest electrocatalytic activity, they scaled up from 2 to 100 cm^2^ with conformal surface morphology on Ni foam and introduced NiFe‐LDH on other conducting cloths and FTO substrate. They demonstrated that electrodeposition is not only a scalable and uniform coating technique but also the applicable tool for diverse substrates. Zhao's group demonstrated the nanostructured MnO_2_ with four different morphologies on carbon fiber paper via the anodic electrodeposition method.^[^ ^40^ ^]^ Distinctive four morphologies (nanosphere, nanosheets, nanoflowers, and nanorods) were achieved by controlling the H_2_SO_4_ concentration and current density. They showed that the same materials could come up with different shapes via the electrodeposition method, and 3D substrates like carbon fiber paper can be uniformly coated.

Nanostructures such as nanorods, nanowires can be uniformly coated with electrodeposits in the form of nanodots, nanoparticles, metal hydroxides, and so on. We have demonstrated that conformal coatings of bismuth vanadate (BiVO_4_) on metal oxide nanorods via pulsed electrodeposition.^[^ ^41^, ^42^ ^]^ By controlling the number of deposition cycles, the coverage of the BiVO_4_ nanoparticles/nanodots were easily controlled on the surface of the nanorods, and core‐shell structures were achieved with optimum deposition condition. In addition, with the two‐step electrodeposition by Yang et al., where the initial deposition was conducted to make nucleation, and the following step was done for the growth of the films, the precursor BiOI films were uniformly deposited on the metal oxide nanorods.^[^ ^43^ ^]^ Macak et al. reported the self‐doping and filling of the TiO_2_ nanotubes via the electrodeposition method.^[^ ^44^ ^]^ They enhanced the low conductivity of as‐formed TiO_2_ nanotube walls by self‐doped Ti^3+^ and conducted the Cu electrodeposition to fill the TiO_2_ nanotubes continuously. By elaborate control of the electrochemical process as a versatile tool, it is possible to create unique nanoscale structures.

Electrodeposition allows achieving area‐selective coatings by exploiting the difference in conductivity of the patterned substrates. When the current or potential is applied to the patterned substrates, the electrochemical reaction occurs at a relatively low resistive area. Zhu et al. reported the self‐charged generator as a power source for electrodeposition. Using a generated electric power, uniform silver microstructures with fine grains were formed on a pattered Au/Si substrates where silver metal is the sacrificial anode and gold surface is the cathode.^[^ ^45^ ^]^ Halpern et al. electrodeposited gold, silver, and nickel on the lithographically nanoring‐patterned substrates.^[^ ^46^ ^]^ Meng's group demonstrated the electrochemical synthesis of copper wires on aminosilane stripe patterns on silicon dioxide substrates.^[^ ^47^ ^]^ The conformal and nanostructural coatings can be applied to not only the flat substrates but also complicated 3D substrates with diverse materials.

## 3 MATERIALS

As mentioned above, conformal coatings with various dimensions and morphologies can be achieved via electrodeposition. Using the same substance, it is possible to synthesize with precisely controlled sizes and morphologies (e.g., nanoparticles having distinctive morphologies ranging from spherical to branch shape) by changing the synthesis conditions. In addition, nanoscale electrodeposition has the advantage of forming almost every element under mild conditions, even the substrates that are difficult to obtain in conventional synthesis and coatings with various compositions can be easily controlled. This section mainly deals with uniformly electrodeposited metal oxides, hydroxides, metal, and metal–organic frameworks (MOFs) and focuses on their morphological/dimensional aspects.

### 3.1 Metal oxides and hydroxides

**FIGURE 4:**
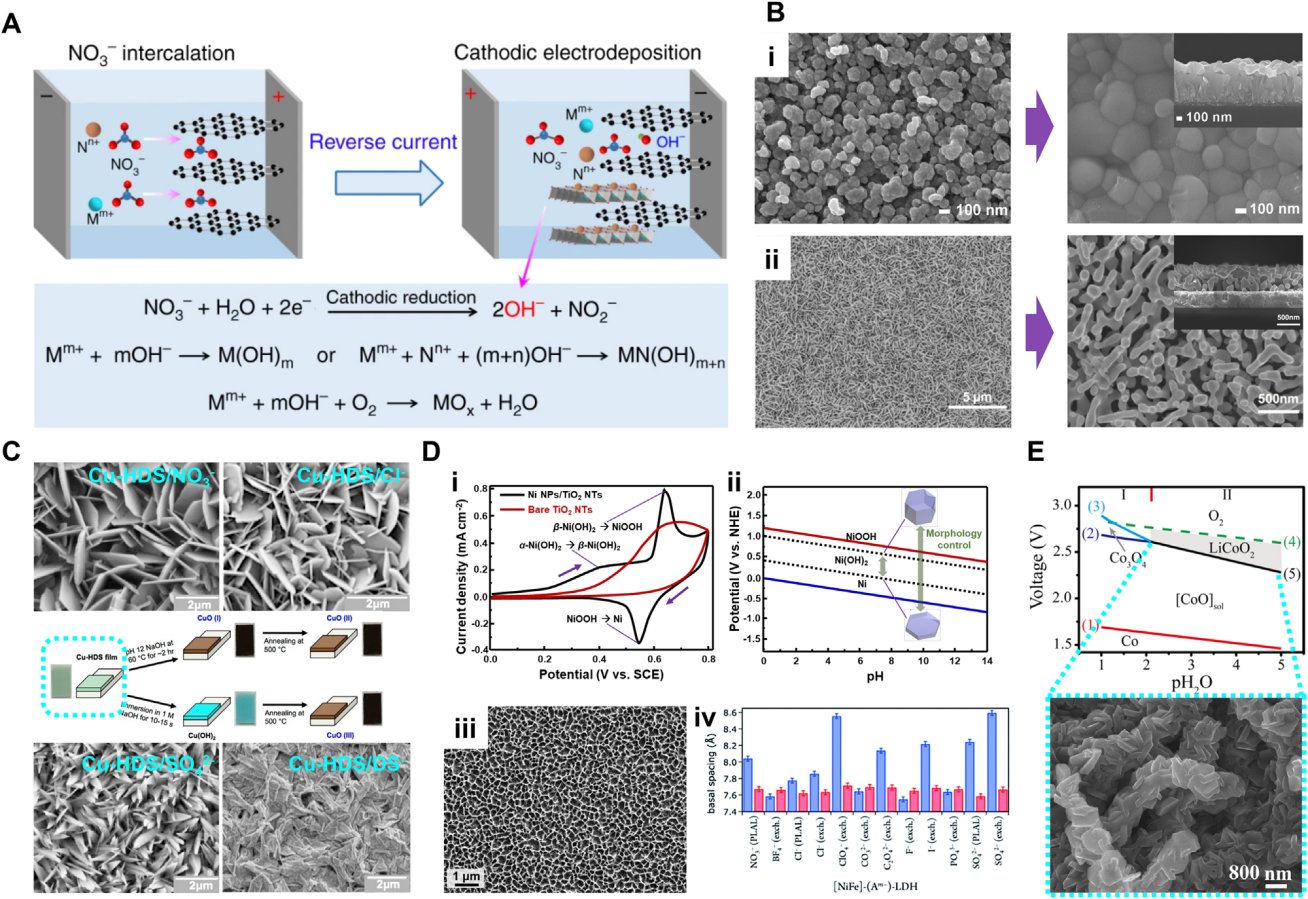
Electrodepositions of metal oxides and hydroxides. (A) The reaction mechanisms of the cathodic electrodeposition of metal hydroxide (M(OH)*_m_* or MN(OH)*_m_*_+_ *_n_*), and oxides (MO*_x_*). Adapted with permission.^[^ ^26^ ^]^ Copyright 2018, Nature Publishing Group. (B(i)) SEM images of the electrodeposited bismuth precursor films (BPF) and BiVO_4_ films after thermal conversion. Adapted with permission.^[^ ^49^ ^]^ Copyright 2018, Wiley‐VCH. (B(ii)) SEM images of the electrodeposited BiOI precursors and nanoporous BiVO_4_ after thermal conversion. Adapted with permission.^[^ ^50^ ^]^ Copyright 2016, Wiley‐VCH. (C) SEM images of Cu hydroxy double salt (HDS) films with different anions. Adapted with permission.^[^ ^51^ ^]^ Copyright 2017, American Chemical Society. (D(i)) The cyclic voltammetry depositions of Ni nanoparticles (NPs) on the TiO_2_ nanotubes (NTs). Adapted with permission.^[^ ^54^ ^]^ Copyright 2017, Elsevier. (D(ii)) Morphology changes of Ni according to the deposition solution conditions. Adapted with permission.^[^ ^56^ ^]^ Copyright 2018, AIP Publishing. (D(iii)) SEM image of the CoO*_x_* nanowall (NW). Adapted with permission.^[^ ^57^ ^]^ Copyright 2020, American Chemical Society. (D(iv)) The basal spacing of the NiFe layered double hydroxides (LDH) with different anions as‐synthesized (blue) and after suspension in KOH (red). Adapted with permission.^[^ ^62^ ^]^ Copyright 2016, Royal Society of Chemistry. (E) pH_2_O‐V diagram of the LiOH‐KOH‐CoO eutectic system and electrodeposited LiCoO_2_ electrodes. Adapted with permission.^[^ ^66^ ^]^ Copyright 2017, AAAS

To synthesize metal oxides or metal hydroxides, the cathodic electrodeposition, where the product is formed on the cathode, is typically used. As shown in Figure 4A, in this process, it is important to select anion species in the plating solution because the cathodic reduction of anion species generates hydroxide ions and increases the local pH around the cathode.^[^ ^26^ ^]^ The increase of the local pH derives the oxidation of metal ions and generates metal oxides (MO*_x_*) or hydroxides (M(OH)*_m_*, MN(OH)*_m_*_+_ *_n_*) on the cathode surface. The applied potential is determined by the standard reduction potentials of anion species such as NO_3_^–^, SO_4_^2–^, ClO_3_^–^, and IO_3_^–^. Various factors such as cation concentration, temperature, pH, and substrate also affect the morphology of the metal oxides and hydroxides. Crystalline metal oxides are also obtained through the post thermal conversion of electrodeposited metal hydroxides.^[^ ^5^, ^48^ ^]^

Even the same material has different morphologies depending on the structure of the electrodeposited precursors. As shown in SEM images of bismuth precursors for BiVO_4_, Bi_2_O_3_ has a nanoparticle structure (Figure 4B(i)),^[^ ^49^ ^]^ and BiOI has a nanoplate structure (Figure 4B(ii)).^[^ ^50^ ^]^ After thermal conversion in soaking vanadium solution, BiVO_4_ films have morphology differences (grain structure and nanoporous structure, respectively). It is mainly originated from the different anion species of NO_3_^–^ and IO_3_^–^, respectively, and accompanying differences in applied potentials affect the oxidation of bismuth ions. The nanostructuring by the cathodic electrodeposition derives the improvement of electrical, optical, and chemical properties.^[^ ^41^, ^42^, ^43^ ^]^

Hydroxy double salts (HDS), where hydroxide ions and other anion species coexist, can also be synthesized by the cathodic electrodeposition. HDS are layered compounds that have a general formula of M^2+^(OH)_2−_ *_x_* (A*^n^* ^–^)*_x_*_/_ *_n_* ∙*y*H_2_O (M: metal ions, A: anion species) have various morphologies according to the anion species. Cardiel et al. made various Cu‐HDS as precursors of CuO photocathodes by using the cathodic electrodeposition.^[^ ^51^ ^]^ The applied potential was used to reduce *p*‐benzoquinone to hydroquinone, elevating the local pH at the cathode.^[^ ^52^, ^53^ ^]^ The pH change triggered the reduction of anion species, making the reduction of Cu^2+^ thermodynamically feasible. As shown in Figure 4C, these chain reactions affect the growth of copper and cause different morphologies of Cu‐HDS depending on the type of the anion species such as NO_3_^–^, Cl^–^, SO_4_^2–^, and dodecyl sulfate ions (DS^–^). The Cu‐HDS/NO_3_^–^ and the Cu‐HDS/Cl^–^ films showed plate‐like crystal structures, and the Cu‐HDS/SO_4_^2–^ films showed bladelike nanocrystal structures. The surfactant effects of DS^–^ made a dense conglomeration of nanoplates of Cu‐HDS/DS^–^.

Transition metal, which has multivalence by the partially occupied d orbital, is synthesized to various phases such as hydroxides and oxyhydroxides depending on the potential.^[^ ^6^, ^26^, ^28^, ^30^ ^]^ As shown in Figure 4D(i), peaks in cyclic voltammogram (CV) were not observed in bare TiO_2_ nanotubes (NTs), whereas Ni nanoparticles (NPs)/TiO_2_ NTs represent the anodic and cathodic peak according to the voltage sweep.^[^ ^54^ ^]^ The oxidation of α‐Ni(OH)_2_ to β‐Ni(OH)_2_ and β‐Ni(OH)_2_ to NiOOH occurred in the forward sweep, and the reduction of NiOOH occurred in the backward sweep. Based on this, it is known that the formation of hydroxides and oxyhydroxides, as well as their morphologies, can be determined by controlling the applied potential. When it is applied to an electrocatalyst, various phases based on transition metals affect the catalytic activity.^[^ ^55^ ^]^ As shown in Figure 4D(ii), this transition is also determined by the solution pH.^[^ ^56^ ^]^ According to the Pourbaix diagram obtained from the first‐principles calculation, it can be seen that the low potential region and high potential region where the Ni(OH)_2_ and NiOOH are stable, respectively, change linearly with pH. Also, depending on potential and pH, the morphology of Ni(OH)_2_ inside the boundaries varies since the stable facet is changed.

In the electrodeposition of the transition metal oxide, morphologies can also be controlled by the kind of additives. Lee et al. electrodeposited two types of amorphous cobalt oxide (CoO*_x_*) on n‐Si by adding different additives in the plating solution.^[^ ^57^ ^]^ As shown in SEM images (Figure 4D(iii)), electrodeposited films using thiourea as an additive, conformal CoO*_x_* nanowalls (NWs) were formed. Whereas, in the solution where boric acid was added as the additive, CoO*_x_* nanoparticles (NPs) were formed. Thiourea, which is used as a leveling and bright agent, promoted the attachment of cobalt ions, causing favorable growth.^[^ ^58^ ^]^ And, boric acid prevented the increase of local pH during the electrodeposition as a buffer agent, inhibiting the formation of hydroxide species.^[^ ^59^, ^60^ ^]^

Layered double hydroxides (LDHs) consisted of Brucite‐like layers with metal cations and hydroxide ions, and interlayers with anion species can also be synthesized by the cathodic electrodeposition.^[^ ^29^, ^61^ ^]^ LDHs are alternative layered compounds that have a general formula of [M^2+^_1−_ *_x_* N^3+^ *_x_* (OH)_2_]*^x^* ^+^[(A*^n^* ^–^)*_x_*_/_ *_n_* ∙*y*H_2_O]*^x^* ^–^ (M, N: metal ions, A: anion species) have various morphologies according to the anion species. As shown in Figure 4D(iv), the basal spacing, which is the thickness of Brucite‐like layers and interlayers, varies according to the size of the anion species.^[^ ^62^ ^]^ When LDHs are used as an electrocatalyst, selecting the anion species in the electrodeposition process is important because the interlayer spacing affects areal capacity and catalytic activity.^[^ ^63^ ^]^ Also, since the concentration of anion species causes the morphology change of LDHs, the additional supply of anion sources should be optimized.

Lithium transition metal oxides (LTMO), widely used in Li‐ion batteries as well as transition metal oxides, can be synthesized by electrodeposition.^[^ ^64^ ^]^ To improve the existing problems such as the inclusion of water, undesired cations, and crystal disorder, various electrodeposition methods have been developed.^[^ ^6^, ^65^ ^]^ As cathode materials of Li‐ion batteries, layered LiCoO_2_, and spinel LiMn_2_O_4_ have been widely used, and dimension control in electrodeposition has importance for smooth intercalation reaction of Li ions. Zhang et al. reported a low‐temperature molten salt electrodeposition methodology, which can obtain highly crystalline LTMO compared with the traditional high‐temperature method.^[^ ^66^ ^]^ Based on the CV analysis, LiCoO_2_ could be electrodeposited at low temperature (260 ℃) in a near‐eutectic mixture of LiOH, KOH, and CoO. As shown in the Pourbaix diagram of LiCoO_2_ (Figure 4E), the eutectic melt of LiOH and KOH is stable in the large potential window and can dissolve well transition metal oxides at low temperatures. The SEM image shows the KOH‐LiOH‐CoO eutectic system can make the conformal electrodeposition of LiCoO_2_ on a porous carbon nanofiber.

### 3.2 Metals

**FIGURE 5:**
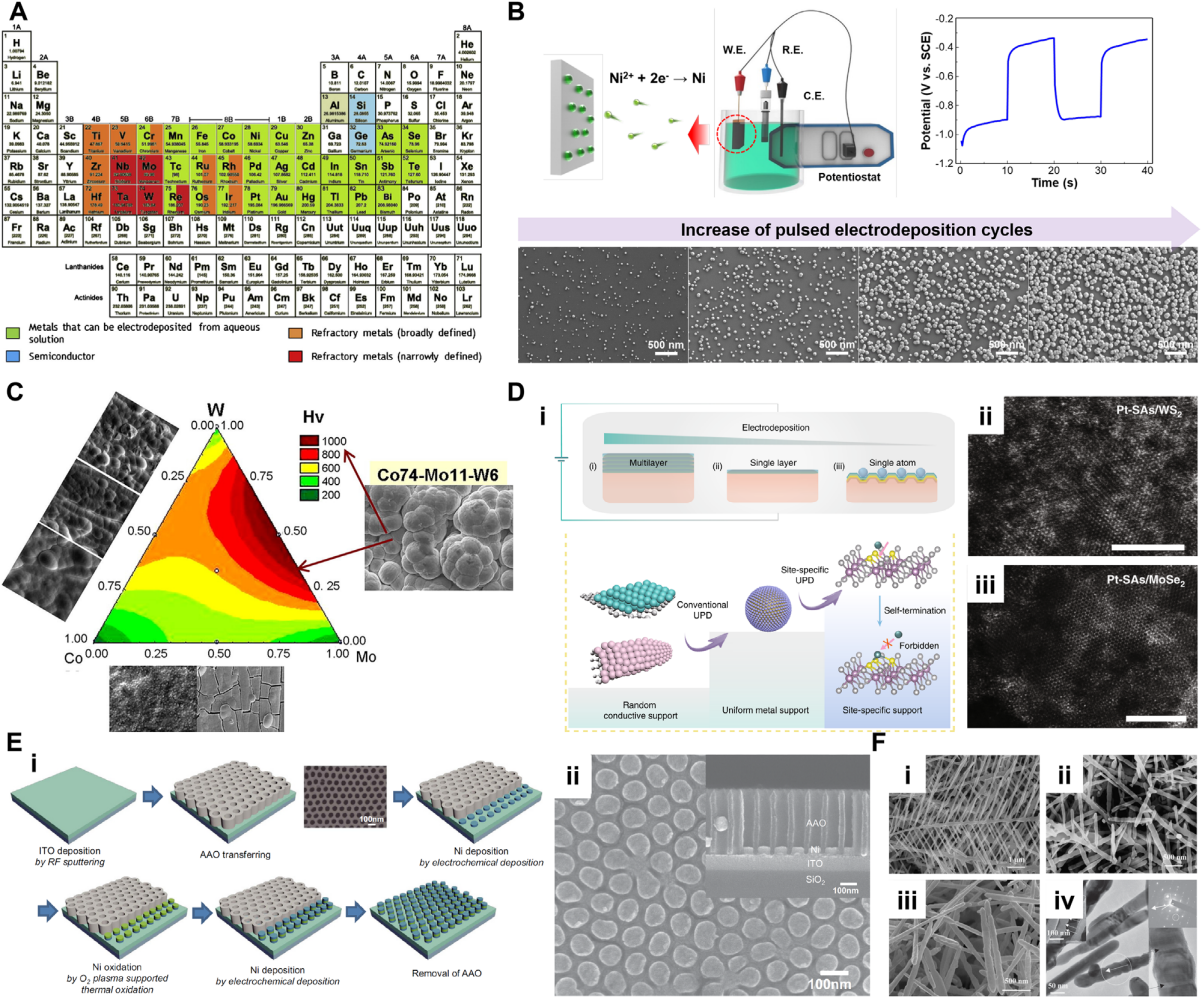
Electrodeposition of metals. (A) Periodic table of metals that can be electrodeposited from an aqueous solution. Reprinted with permission.^[^ ^67^ ^]^ Copyright 2015, Elsevier. (B) Schematics of pulsed electrodeposition of Ni nanoparticles (NPs) and SEM images with Ni NPs on n‐Si with various numbers of pulsed deposition cycles. Reprinted with permission.^[^ ^70^ ^]^ Copyright 2018, American Chemical Society. (C) Surface morphologies and microhardness distributions of the electrodeposited ternary Co‐Mo‐W alloys. Reprinted with permission.^[^ ^72^ ^]^ Copyright 2018, Elsevier. (D(i)) Methodological development of atomically dispersed metal catalysts (ADMCs) preparation by electrodeposition. Magnified high‐angle annular dark‐field‐scanning transmission electron microscopy (HAADF‐STEM) images of (D(ii)) Pt‐single atoms (SAs)/WS_2_ and (D(iii)) Pt‐SAs/MoSe_2_ (scale bars: 5 nm). Reprinted with permission.^[^ ^38^ ^]^ Copyright 2020, Nature Publishing Group. (E(i)) Schematic procedure for nanoscale Ni‐NiO‐Ni arrays with AAO templates. (E(ii)) Planar and cross‐sectional SEM images of electrochemically deposited Ni nanodots. Reprinted with permission.^[^ ^81^ ^]^ Copyright 2016, Nature Publishing Group. SEM images of silver nanowires with relaxation times of (F(i)) 0 min, (F(ii)) 30 min, and (F(iii)) 120 min. (F(iv)) TEM images of silver nanowires with relaxation time of 120 min. Reprinted with permission.^[^ ^85^ ^]^ Copyright 2009, Royal Society of Chemistry

In general, metals are cathodically electrodeposited by reducing metal ions according to the standard reduction potential in aqueous solutions. As shown in the Pourbaix diagram of nickel (Figure 4D(ii)), metal phases are stably deposited from the aqueous solution when further reduction of adsorbed ions occurs depending on the electrode potential and pH.^[^ ^56^ ^]^ As shown in the periodic table (Figure 5A), a wide range of elements (green) can be electrodeposited from the aqueous solutions.^[^ ^67^ ^]^ Also, some elements are difficult to synthesize electrochemically in aqueous systems, such as Al and refractory metals (red, orange). However, these metals can be electrodeposited from ionic liquids organic solvents. Also, through the galvanic deposition, where the substrate is immersed into solution without potential sources, noble and refractory elements can be easily synthesized from the substrate where electrons are spontaneously supplied.^[^ ^68^, ^69^ ^]^

Pulse electrodeposition is a widely used method for synthesizing metal particles because it can make a uniform size distribution by suppressing the concentration gradient during the pulse‐off time. Lee et al. controlled the coverage of Ni NPs on the Si by modulating the pulse cycles in galvanostatic pulsed electrodeposition.^[^ ^70^ ^]^ As shown in Figure 5B, the nucleation and growth rate of metal ions are affected by variables such as on/off time, applied current, and pulse cycle, and Ni NPs were conformally coated without agglomeration under optimal conditions. Sun et al. uniformly deposited copper‐doped nickel cubic nanopore on the Ni foam by using a potentiostat pulse electrodeposition.^[^ ^71^ ^]^ Based on the method, adhesion between Cu‐doped Ni and Ni foam was increased due to narrow pulse width, high frequency off current, and reduction of concentration polarization.

Metal alloys can be electrodeposited by reducing metal ions together in which standard reduction potential windows are overlapped. Mukhamedova et al. electrodeposited binary and ternary alloys of cobalt with molybdenum and tungsten.^[^ ^72^ ^]^ Mo and W, which are representative refractory metals, were alloyed with Co through pulsed electrodeposition in the biligand citrate‐pyrophosphate electrolyte without temperature rise. Binary Co‐Mo and Co‐W alloys were well deposited without micro‐cracks, and ternary Co‐Mo‐W alloy had a smoother surface than binary alloys. As shown in Figure 5C, it is possible to check the microstructure of alloys in the ternary composition diagram. Also, the microhardness of alloys varied according to the microstructural and compositional changes, and the microhardness improved as the content of refractory metals increased. In particular, electrodeposition of alloys is actively used to find new compositions in the electrocatalyst field. Since it is a solution‐based process, close control is easier than the vacuum process, and ternary and quarternary diagrams with more specific compositions can be completed. Cao et al. reported combinatorial electrodeposition to synthesize mixed‐metal selenides for oxygen evolution (OER) catalyst.^[^ ^73^ ^]^ The quarternary mixed‐metal selenides were based on ternary Ni‐Fe‐Co transition metal alloy, and an equimolar mixture of the metal sulfate precursors and selenium sources was used. Through the electrochemical measurement for each composition, the catalytic performances when the ternary Ni‐Fe‐Co alloy was combined with Se could be expressed in a compositional phase diagram. By compactly controlling the compositions of electroplating solutions, it was found that the optimal (Ni_0.25_Fe_0.86_Co_0.07_)_3_Se_4_ is the electrocatalyst with the lowest overpotential.

High entropy alloys (HEAs), which are formed by mixing five or more metal elements with identical proportions, can also be made by using the electrodeposition method. Unlike traditional alloys, large mixing entropies decrease the free energy of the system and make the solid solutions stable phases. Yoosefan et al. firstly made CoCrFeMnNi HEAs via the pulse electrodeposition method, and a single solid solution structure of face‐centered cubic was identified by the XRD analysis.^[^ ^74^ ^]^ The crystalline size of HEAs decreases according to the increase of both pulse cycle and frequency. Also, Soare et al. reported AlCrFeMnNi and AlCrCuFeMnNi high entropy alloys prepared by the potentiostatic electrodeposition.^[^ ^75^ ^]^ These HEAs were co‐deposited in an electrolyte based on a DMF‐CH_3_CN organic compound. The non‐aqueous solvent is suitable for the electrodeposition of HEAs because of their good chemical stability and high electrical conductivity.

Atomically dispersed metal catalysts (ADMCs), which minimize the size of noble metals and maximize atomic efficiency, have been actively studied in the catalysis field.^[^ ^76^, ^77^ ^]^ Although the electrodeposition is simple and efficient in synthesizing the ADMCs, it has the disadvantage of accompanying inevitable bulk products. As shown in Figure 5D(i), the general electrodeposition of metals results in the formation of inevitable multilayered bulk phases.^[^ ^38^ ^]^ And, to scale down metal thin films to a single layer, conventional UPD, by which monolayers are formed at potential above the thermodynamic value, have been used. Shi et al. devised site‐specific electrodeposition on transition metal dichalcogenides (TMDs) with isolated active sites for UPD to synthesize the ADMCs. During the UPD process on the TMDs, metal–support interaction is more energetically favorable than metal–metal interaction because of the lone pair electrons and suitable electronegativity of chalcogen sites. As a result, the site‐specific UPD makes a single atom growth self‐terminated, diminishing the aggregation of metal atoms. Single Pt atoms were underpotentially deposited on various TMDs such as WS_2_, MoSe_2_, WSe_2_, and MoTe_2_. As shown in Figure 5D(ii,iii), the high‐angle annular dark‐field‐scanning transmission electron microscopic images show conformally distributed single Pt atoms on the WS_2_ and MoSe_2_ support, respectively.

As shown in schematics (Figure 5E(i)), by the introduction of the anodic aluminum oxide (AAO), well‐ordered 1D nanostructures can be easily obtained using electrodeposition.^[^ ^78^, ^79^, ^80^ ^]^ Based on structural advantages, mechanical stability, and insulating properties, AAO is used as a template for the electrodeposition of various materials that are difficult to grow anisotropically. Song et al. transferred the AAO template on the ITO substrates and electrochemically synthesized Ni nanodots with the AAO template mask.^[^ ^81^ ^]^ As shown in Figure 5E(ii), Ni nanodots were well‐formed inside the AAO, and through additional thermal oxidation and electrodeposition, Ni/NiO/Ni structured nanodot structures were fabricated. Wang et al. fabricated AAO templates with controlled pore size using the anodizing of Al and transferred it onto Au film.^[^ ^82^ ^]^ In a similar procedure to Ni nanodots, uniform Ni nanorod arrays were also electrodeposited between AAO templates by controlling the deposition time. Various nanostructures of metals can also be fabricated by the template‐free electrodeposition.^[^ ^32^, ^83^, ^84^ ^]^ Fang et al. electrodeposited dendritic Ag nanowires (NWs) and grew into Ag nanorods by the relaxation process without additives.^[^ ^85^ ^]^ A strong DC potential of 30 V was applied between two silver plate electrodes, and the relaxation process in AgNO_3_ solution was used for the elimination of Ag branches. As shown in SEM images, the initial product has dendritic structures (Figure 5F(i)), and, after relaxation, the sub‐branches of dendrites break up and make Ag nanorods (Figure 5F(ii,iii)). During the electrochemical process, Ag dendrites underwent Ostwald ripening, and the following Ag nanorods had bamboo structures, as shown in the TEM image (Figure 5F(iv)). Park et al. vertically grew Ag NWs with diameters in the range of 80 to 800 nm.^[^ ^86^ ^]^ The diameters of NWs were varied by the repetition of nucleation and dissolution in ultra‐dilute electrolytes. Repeated oxidation and reduction potentials determined the size of nucleation islands, where anisotropic nanowires grew up.

### 3.3 MOFs

**FIGURE 6:**
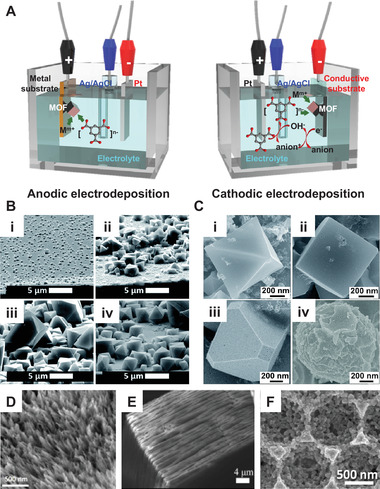
Electrodeposition of metal–organic frameworks (MOFs). (A) Mechanisms of anodic electrodeposition and cathodic electrodeposition of MOFs. (B) SEM images of anodic electrodeposited Cu‐BTC (HKUST‐1) MOFs with different deposition times: (i) initial nucleation, (ii) growth of islands, (iii) intergrowth, and (iv) detachment. Reprinted with permission.^[^ ^91^ ^]^ Copyright 2016, Royal Society of Chemistry. (C) SEM images of cathodic electrodeposited HKUST‐1 MOFs with different deposition potentials: (i) −0.5 V, (ii) −1.0 V, (iii) −1.5 V, and (iv) −2.0 V vs. Ag/AgCl. Reprinted with permission.^[^ ^92^ ^]^ Copyright 2019, Royal Society of Chemistry. (D) SEM image of Co(Ⅱ)‐MOF nanofiber array on Ni foam. Reprinted with permission.^[^ ^93^ ^]^ Copyright 2018, Elsevier. (E) SEM image of Co‐Ni mixed MOFs on Ni foam. Reprinted with permission.^[^ ^96^ ^]^ Copyright 2017, Elsevier. (F) SEM image of HKUST‐1 MOFs on the walls of the ordered macroporous Cu electrodes. Reprinted with permission.^[^ ^99^ ^]^ Copyright 2016, Royal Society of Chemistry

Both anodic electrodeposition and cathodic electrodeposition are used to synthesize the MOFs, which comprise metal centers and organic linkers.^[^ ^87^, ^88^ ^]^ As illustrated in Figure 6A, in the anodic electrodeposition system, metal substrates such as Ni foam and Cu plate are used as ionic sources, which react with organic linkers dissolved in the plating solutions. In more detail, metal ions are released in the solution, and nucleation first occurs at the metal substrates above the critical concentration of metal ions and organic linkers. As the deposition time increases, the nuclei grow further to form an intergrown layer, and the large MOFs are repeatedly detached. On the other hand, in the cathodic electrodeposition system, conducting substrates such as carbon nanofibers are used as a cathode, and both metal ions and organic linkers are contained in the plating solution.^[^ ^89^ ^]^ Firstly, the anion species in the solution are reduced, generating hydroxide ions. Increased pH by the hydroxide ions causes the reaction with organic linkers and deprotonates them. Finally, metal ions are attracted to the cathode, and the deprotonated linkers trigger the assembly with metal ions for the nucleation of MOFs.

HKUST‐1 (Cu‐BTC), where the building block is consisted of Cu ions and trimesic acid, is representative MOFs that can be electrochemically grown.^[^ ^90^, ^91^, ^92^ ^]^ The HKUST‐1 has been synthesized by both the anodic electrodeposition and cathodic electrodeposition. In the case of anodic electrodeposition, the surface defects of substrates and deposition time controls are critical variables for optimal structures of MOFs, and the introduction of pulse mode can precisely control the nucleation and growth rate. Campagnol et al. synthesized HKUST‐1 on the Cu meshes by using anodic electrodeposition and established its mechanism consisting of four phases: nucleation, growth of islands, intergrowth, and detachment.^[^ ^91^ ^]^ As shown in Figure 6B(i), isolated nearly monodispersed nanocrystals were nucleated on the Cu substrate. Since the current is concentrated near the generated nuclei, new nuclei are formed and grown around the MOF crystal (Figure 6B(ii)). Then, the compact layers were formed as nucleation and growth extend to uncovered regions due to increased Cu^2+^ ions (Figure 6B(iii)). In order to obtain high‐quality MOF films, electrodeposition should be terminated at the intergrowth phase. As the last phase (Figure 6B(iv)), detachment of MOFs occurred because the Cu substrate under the MOFs was dissolved by forming Cu ions and creating voids between MOFs/substrate interfaces.

In the cathodic system, the type of anion species and the applied potential are important variables to control the microstructures of MOFs. Li et al. synthesized HKUST‐1 MOFs by using the cathodic electrodeposition.^[^ ^92^ ^]^ As shown in SEM images (Figure 6C(i–iv)), depending on the applied potentials, the morphologies of HKUST‐1 MOFs significantly changed to octahedral, cubic, polyhedral, and random structures. This means that the local pH was changed with the applied potential changes, making the thermodynamically stable surfaces changed. Also, the applied potential change enables the composition and crystal size control of MOFs because it causes the concentration changes of metal cations and organic linkers. Using this, Campagnol et al. devised the cathodic electrodeposition with a step‐like potential that applies different potentials according to time interval.^[^ ^91^ ^]^ At the low potential region, since the concentration of organic linkers relatively decreased compared to metal cations, small crystals and Cu were formed, which improved adhesion to the substrate. The crystal size increased at the high potential region. The gradual morphology can be applied to optimize the catalytic properties of MOFs.

The electrochemical synthesis can facilitate dimension control of MOFs such as 1D, 2D, and 3D. As shown in Figure 6D, vertically aligned 1D nanofiber Co‐MOF arrays are uniformly covered on the Ni foam by using nitrate ions and cyanuric acid as anion species and organic linker, respectively.^[^ ^93^ ^]^ Also, Caddeo et al. synthesized Cu‐based MOFs such as HKUST‐1 and Cu(INA)_2_ (i.e., INA = isonicotinic acid) using template‐assisted electrodeposition.^[^ ^94^ ^]^ They electrodeposited 1D Cu nanowires in the polycarbonate (PC) membrane template and conducted electrochemical oxidation in the solution with organic linkers to convert to 1D MOFs. According to the diameters of PC templates, morphologies of 1D Cu MOFs could be controlled. Hou et al. synthesized template‐free 1D Cu‐based MOFs.^[^ ^95^ ^]^ By using the combination of electrodeposition and hydrothermal synthesis, Cu CAT (i.e., CAT = catecholate) nanowires were synthesized on the self‐supported polypyrrole membrane. The Cu MOFs nanowire arrays structurally have high conductivity and active surface area. Zhang et al. anodically electrodeposited Co‐Ni mixed MOFs on the Ni foam.^[^ ^96^ ^]^ M(HBTC)(DMF)_2_ precursor, which has a 2D layered structure, were used to make building blocks. The intermolecular short contact interactions between DMF and carboxylate group make layered stacks of Co and Ni. As shown in Figure 6E, the MOF crystals were formed by the layer‐by‐layer stacking of 20 nm thick. Also, Huang et al. electrochemically synthesized 2D MOFs from the 3D layered MOFs.^[^ ^97^ ^]^ 3D pillared‐layer Cd_2_(DHBDC) (i.e., DHBDC = 2,3‐dihydroxy‐1,4‐benzenedicarboxylic acid) MOFs firstly were synthesized, and the potentiostatic method made the exfoliation of the 3D MOFs. Pillars were oxidized to lower coordination ability and kept the transformation to 2D structures. Also, Bradshaw's group reported the electrochemical synthesis of 2D Cu_3_(HHTP)_2_ MOFs (i.e., HHTP = hexahydroxytriphenylene) from the Cu metal layer substrate.^[^ ^98^ ^]^ The formation of Cu_3_(HHTP)_2_ was conducted through the dissolution of the Cu layer and in combination with the HHTP ligand by applying the anodic potential.

To enhance the specific surface area of MOFs, 3D templates were used as the substrate. As shown in Figure 6F, Warakulwit et al. electrochemically fabricated 3D hierarchically structured HKUST‐1 MOFs on the SiO_2_ colloidal templates made by the Langmuir‐Blodgett method.^[^ ^99^ ^]^ The inverse opal structures can dramatically increase the specific surface area by controlling the applied potential and deposition time, improving the catalytic properties of MOFs. Sablowski et al. also synthesized superhierarchical 3D Cu BTC MOFs on metal substrates.^[^ ^100^ ^]^ The meso/microporous‐structured MOFs crystals were electrochemically triggered, and superhierarchical films were rapidly formed without any templates.

## 4 APPLICATIONS

We have introduced that conformal coatings with a wide variety of phases and shapes can be formed on conductive substrates through nanoscale electrodeposition. The electrochemical synthesis technology has been developed to uniformly introduce target materials into substrates from 3D to 0D, suggesting that, unlike conventional bulk electrodeposition methods, nanoscale electrodeposition is highly precise and can also be used in nanoscale applications. In fact, nanoscale electrodeposition is used in a wide range of applications such as sensors, supercapacitors, and so on. Particularly, energy conversion applications adapted with nanoscale electrodeposition reports high properties and efficiencies. In this chapter, we selectively introduce the research trends and influences of nanoscale electrodeposition in the field of Li electrodes for batteries, water splitting photoelectrodes, and electrocatalysts.

### 4.1 Li electrodes for batteries

Lithium (Li), the crucial material for the future energy storage system, can be effectively produced through the electrodeposition method. Li‐based batteries induced by electrodeposition can have the virtues of high uniformity and facile controllability of film, leading to improved battery performance and stability. The existing hindrance in Li formation, such as unstable solid‐electrolyte interphase (SEI), poor cycling lifetime, low efficiency, dendrite formation, and inhomogeneous morphology, can also be efficiently resolved through electrodeposition engineering.^[^ ^64^, ^101^, ^102^, ^103^ ^]^

Figuring out the nucleation and morphology formation mechanisms during Li electrodeposition is crucial for developing high‐performance Li batteries. Fine control of Li growth and distribution can be possible through various chemicals, substrates, and electrolytes conditions. Pei et al. experimentally revealed the morphology change of Li on a copper substrate during the initial electrodeposition nucleation stage.^[^ ^12^ ^]^ They found that the size of Li nuclei is proportional to the inverse of overpotential and the number density of Li nuclei is proportional to the cubic power of overpotential. Through utilizing researched electrochemical correlation, enhancement in uniformity of Li film could be achieved during galvanostatic electrodeposition. Hao et al. also reported the morphology dependence of Li electrodeposition on the reaction kinetics (overpotential), electrolyte transport, and substrate.^[^ ^104^ ^]^ They found that Li film porosity is strongly governed by Li self‐diffusion kinetics and fast ion diffusion in electrolytes contributes to homogeneous deposition. As for the substrate effect, the surface protrusion is the key spot for the initiation of Li dendrite since metallic Li preferentially deposits over the surface protrusion at a high reaction rate, resulting in the generation of Li dendrite.

Based on the understanding of electrochemical Li growth, there has been noteworthy progress in the regulation of Li electrodeposition. One of the strategies for stable and efficient Li growth is finding proper electrolytes and additives. Controlling the growth of dendrite and SEI formation can be possible through optimizing electrolytes or additives conditions.^[^ ^105^, ^106^, ^107^, ^108^ ^]^ Along with conventional liquid‐type electrolyte, solid or gel‐type electrolyte like solid polymer electrolyte, electrolyte reinforced with halogenated lithium salts which have high mechanical strength, are consistently researched due to their role of stabilization in battery cycling performance.^[^ ^106^, ^109^, ^110^, ^111^ ^]^ Additives in electrolytes are also known to have various effects in Li electrodeposition. Lithium polysulfide, lithium nitrate, lithium fluoride, and ions like Cs^+^, Rb^+^ are revealed to assist the uniform deposition and effective control of SEI formation. Developing lithiophilic anodic hosts or artificial SEIs are also being researched for the further stable growth of Li batteries.^[^ ^105^, ^112^, ^113^, ^114^ ^]^

**FIGURE 7:**
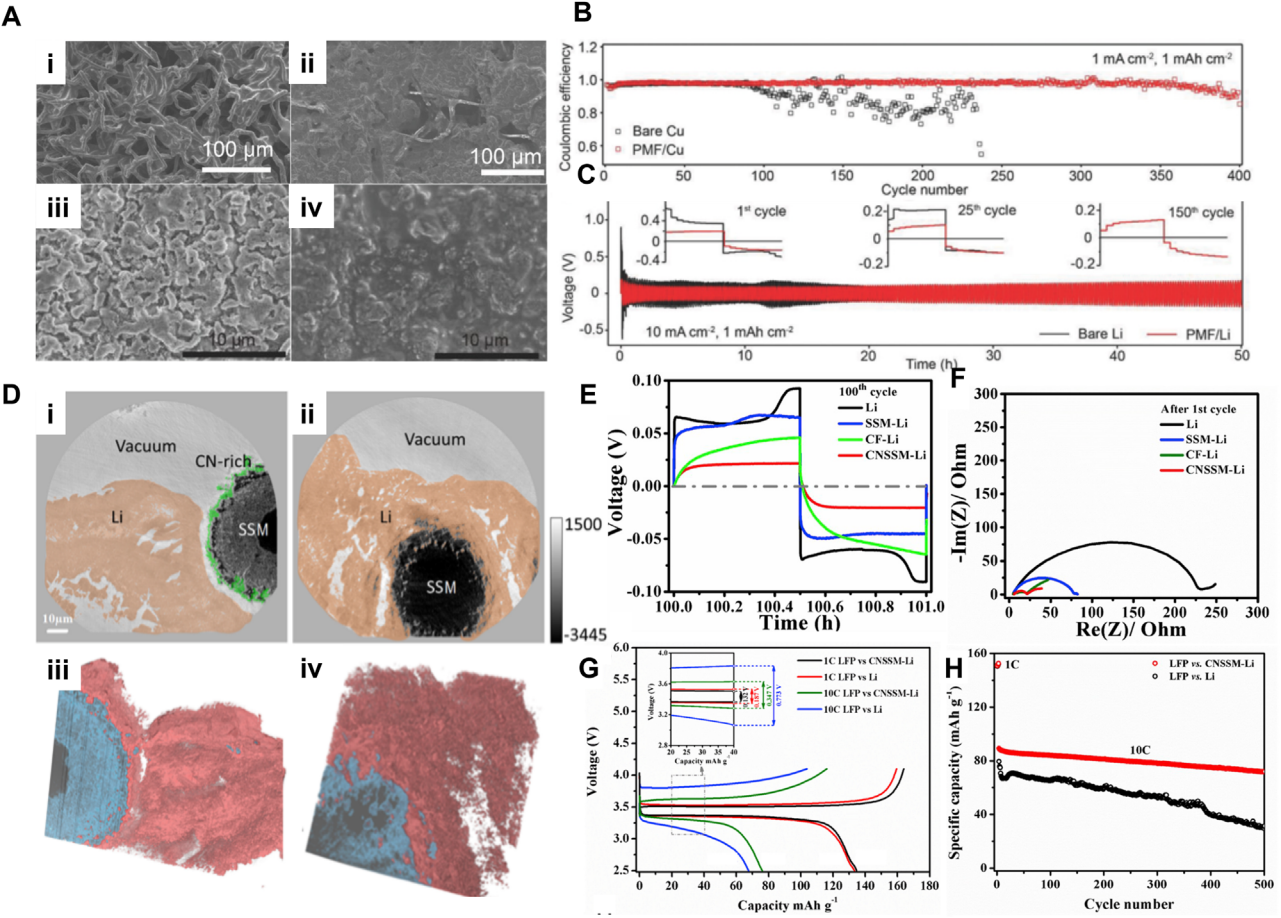
(A) SEM images of (i) bare Cu foil after depositing lithium 1 mAh cm^–2^ at a current density of 1 mA cm^–2^, (ii) Cu foil with 3D PMF host after depositing lithium 1 mAh cm^–2^ at a current density of 1 mA cm^–2^, (iii) Lithium electrodeposited for tenth cycle (1 mA cm^–2^, 1 mAh cm^–2^) on bare Cu, and (iv) Lithium electrodeposited for tenth cycle (1 mA cm^–2^, 1 mAh cm^–2^) on PMF/Cu. (B) Lithium Coulombic efficiency measurements for Li/Cu cells with or without the protection of 3D PMF host at 1 mA cm^–2^. (C) Voltage profile of bare lithium electrodes of 3D PMF/Li composite electrodes at current density 10 mA cm^–2^ with the capacity of 1 mAh cm^–2^. (A–C) Adapted with permission.^[^ ^124^ ^]^ Copyright 2018, Wiley‐VCH. (D) Virtual cross‐sections from X‐ray holographic nano‐tomography. (i) a pristine CNSSM‐Li composite, (ii) a CNSSM‐Li composite after 2 h of Li electrodeposition at 1 mA cm^−2^. Rendering of the 3D tomographic reconstructed results from (iii) pristine CNSSM‐Li composite, and (iv) CNSSM‐Li composite after 2 h of Li electrodeposition at 1 mA cm^−2^. (E) Voltage profiles of the 100^th^ cycle. (F) Nyquist plots of the symmetrical cells after the first cycle. (G) Voltage profile comparison of the LFP||Li cells and LFP||CNSSM‐Li cells at 1 C (1C  =  167 mA g^−1^) (nominal current  =  0.334 mA cm^−2^) and 10 C; the hysteresis in the voltage profile is enlarged and shown in the inset. (H) Cell cycling performance of LFP paired with Li and CNSSM‐Li composite electrode. The mass loadings of LFP, LMO, and NCM622 cathode are ∼2 mg cm^−2^. (D–H) Adapted with permission.^[^ ^115^ ^]^ Copyright 2020, Elsevier

A recent emerging strategy for electrodepositing high‐performance Li batteries is introducing an effective host substrate. It has been found that due to the inhomogeneous morphology of lithium or unstable SEI state, locally concentrated lithium ion flux appears at the electrode‐electrolyte interface, leading to the acceleration of surface dendrite. This causes the local high current densities and volume expansion which can deteriorate the cyclic performance of the Li battery. The 3D composite structure can act as a high surface area current collector, which can derive superior electrochemical performance through solving the above problems and reduce interfacial resistance in Li electrodeposition.^[^ ^64^, ^105^, ^107^, ^114^, ^115^, ^116^ ^]^ 3D scaffolds like 3D porous Cu foil, 3D carbon nanofiber, Li‐coated 3D polymeric matrix, Li‐carbon nanofiber, and Li‐carbonized wood are continuously researched for effective Li growth substrate.^[^ ^117^, ^118^, ^119^, ^120^, ^121^, ^122^, ^123^ ^]^ Fan et al. demonstrated a stable Li metal battery through a 3D porous poly‐melamine‐formaldehyde (PMF) host.^[^ ^124^ ^]^ Numerous polar groups (amine and triazine) in nonconductive 3D PMF can efficiently regulate Li‐ions distribution, leading to uniform and Li dense film morphology. Figure 7A(i,ii) shows SEM images of Li deposited on bare Cu and PMF/Cu. Li on bare Cu shows dendrite with needle‐like structure while those dendrites are eliminated in Li on PMF/Cu. After 10 cycles of operation, Li cell using PMF/Cu (Figure 7A(iv)) retained its structure well compared to Li cell using bare Cu (Figure 7A(iii)). Lithium coulombic efficiency measurement of Li cell (Figure 7B) and cycling performance of symmetrical cells (Figure 7C) with and without the protection of 3D PMF host reveal the battery performance enhancement through using effective 3D substrates. In addition to 3D nonconductive substrate, He et al. introduced a conductive lithiophilic carbon‐nitrogen modified stainless steel mesh host that can induce ionically conductive and uniform lithium surface.^[^ ^115^ ^]^ Figure 7D(i–iv) shows virtual cross‐sections reconstructed from the nano‐tomographic measurements and images. They indicate that Li is mainly electrodeposited around the CNSSM, resulting in homogeneous morphology and the lower nucleation overpotential. Voltage profiles (Figure 7E,G), Nyquist plots (Figure 7F), and cyclic cell performance (Figure 7H) reveal that Li on CNSSM structure has excellent electrochemical battery performance with largely improved stability and low hysteresis.

### 4.2 Photoelectrodes

**FIGURE 8:**
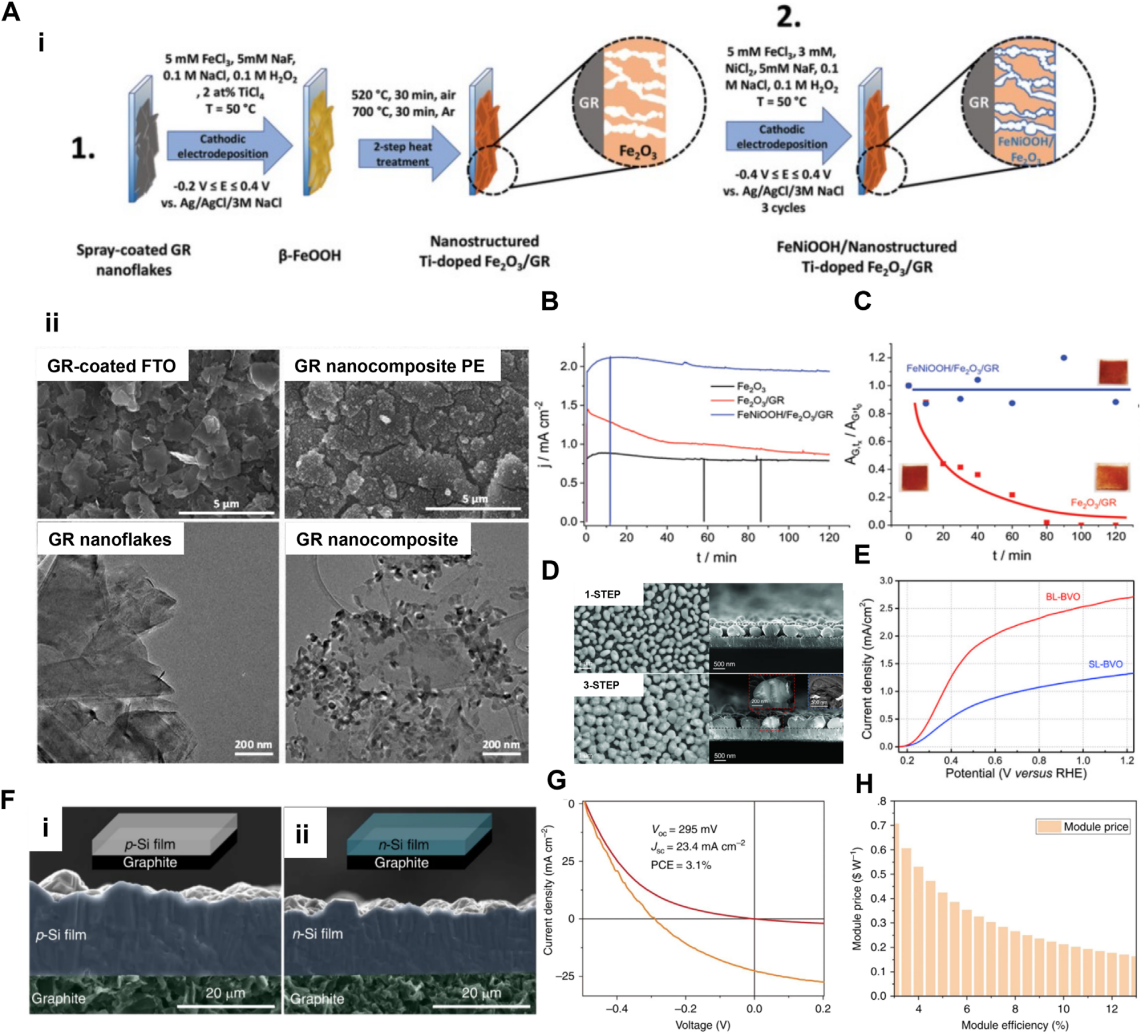
(A(i)) Schematic illustration of synthesis methods applied to prepare the nanohybrid electrode samples: 1) cathodic electrodeposition of Fe_2_O_3_ and 2) surface decoration with FeNiOOH co‐catalyst. (A(ii)) SEM images captured for GR‐coated FTO, Fe_2_O_3_/GR nanocomposite photoelectrode (QED = 450 mC cm^–2^) and TEM images taken for GR nanoflakes, Fe_2_O_3_/GR nanocomposite (QED = 450 mC cm^–2^). (B) Long‐term chronoamperometry measurements performed in 1 M NaOH solution saturated with Ar, applying E = 1.45 V versus RHE potential and under 100 mW cm^–2^ AM 1.5 simulated sunlight. The vertical lines show the current drop upon interrupting the illumination. (C) Change in the normalized area of the G band of GR in time. (A–C) Reprinted with permission.^[^ ^141^ ^]^ Copyright 2020, Wiley‐VCH. (D) SEM images of the one‐step prepared BL‐BVO and the three‐step prepared BL‐BVO films (the insets are the high magnification SEM and TEM image). (E) Photocurrent density–voltage (*J*–*V*) curves under AM 1.5G illumination (100 mW cm^−2^) of BVO photoanodes in 0.1 m K_2_B_4_O_7_·4H_2_O with 0.1 m Na_2_SO_3_ (pH = 9.52). (D,E) Reprinted with permission.^[^ ^135^ ^]^ Copyright 2018, Wiley‐VCH. (F) SEM images of (i) p‐type and (ii) n‐type silicon film on the graphite substrate. (G) Solar cell devices based on the electrodeposited p‐type silicon films. The devices are tested under dark (red line) and 100 mW cm^−2^ illumination, respectively. (H) Cost analysis based on molten salt electrodeposition technique. The manufacturing cost of the solar cell module per watt is plotted versus module efficiency. (F–H) Reprinted with permission.^[^ ^150^ ^]^ Copyright 2019, Nature Publishing Group

Uniform and finely controlled semiconductor or metallic films for a photovoltaic application like solar cells, water splitting photoelectrodes are consistently researched. Electrochemically designed films exhibit high photoactivity by facile light absorption and charge separation.^[^ ^125^, ^126^, ^127^, ^128^ ^]^ Semiconductor films like Fe_2_O_3_, TiO_2_, BiVO_4,_ and Cu_2_O are widely utilized in photoelectrochemical (PEC) water splitting electrodes due to their high light‐harvesting ability.^[^ ^129^, ^130^, ^131^, ^132^, ^133^, ^134^, ^135^, ^136^ ^]^ Facile control of thickness and morphology through the electrodeposition method can effectively adjust the dynamics of film appropriate for boosting PEC performance. For instance, Lee et al. reported the effect of electrodeposited NiO*_x_*/Ni nanoparticles coverage and crystallinity on the PEC characteristics of NiO*_x_*/Ni/n‐Si photoanodes. As the number of a cycle in pulsed electrodeposition increased, the coverage of NiO*_x_*/Ni/n‐Si increased. PEC property was enhanced at a certain cycle (fourth), but it gradually degraded after the optimized coverage amount due to the shading effect of nanoparticles at a high coverage state. Also, annealed Ni nanoparticles catalysts exhibited higher PEC characteristics than as‐deposited ones by the formation of NiO*_x_*, which generated a change in the flat band potential.^[^ ^70^ ^]^ However, most of the single semiconductor film has small charge carrier diffusion length, bulk recombination, and sluggish water oxidation/reduction kinetics.^[^ ^128^, ^137^, ^138^, ^139^ ^]^ In order to overcome these issues, controlling film morphology, fabricating heterojunctions, and depositing catalysts have proceeded through electrochemical methods. Fe_2_O_3_ has been extensively studied as photoanode material for its appropriate valence band position to expedite water oxidation, narrow band gap (2.0–2.2 eV), and virtue of earth abundance.^[^ ^126^, ^130^, ^136^ ^]^ Due to the short carrier diffusion length of hematite, nanostructuring of the hematite structure has been intensively studied. Cai et al. demonstrated the hollow nanostructured Sn‐doped hematite water splitting photoanodes synthesized by using template‐assisted electrodeposition and heat‐treatment process. Potential sweep rate in electrodeposition affected the morphology of photoanode with enhancing the entire PEC activities in structure.^[^ ^140^ ^]^ Kormanyos et al. fabricated and investigated PEC performance of hybrid structure of FeNiOOH/Fe_2_O_3_/Graphene (GR) photoelectrode.^[^ ^141^ ^]^ Electrodeposition makes it possible to combine three components structurally and morphologically appropriate for high photocurrent generation. They revealed that Fe_2_O_3_ was responsible for light absorption, GR substrate provided good charge transport, and FeNiOOH overlayer suppressed charge recombination through surface passivation. Figure 8A(i) shows schematics of the synthesis process of FeNiOOH/Fe_2_O_3_/GR photoelectrode. Three steps were proceeded as spray coating of GR on FTO, electrodeposition of β‐NiFeOOH on GR, and two‐step heat treatment of β‐FeOOH/GR to yield Fe_2_O_3_/GR. Figure 8A(ii) shows SEM images of GR‐coated FTO and Fe_2_O_3_/GR nanocomposite photoelectrode and TEM images of GR nanoflakes and Fe_2_O_3_/GR nanocomposite. Figure 8B shows long‐term chronoamperometry measurements to investigate photostability. From the experiments, the photocurrent decrease was assumed due to the degradation of the substrate GR, and this state was elucidated through the periodical record of Raman spectra and change in the normalized area of the G band of GR in time (Figure 8C).

Monoclinic bismuth vanadate (BiVO_4_, BVO) is also rising as efficient photoanode material by its characteristics of good stability in electrolytes, appropriate band structure, and well applicability.^[^ ^136^, ^142^, ^143^ ^]^ Huang et al. reported high‐performance twin structured BVO film‐based photoanode synthesized by electrodeposition.^[^ ^135^ ^]^ Through combining methods of electrodeposition of BiOI nanosheets, thermo‐chemical conversion to prepare BVO films with twin structure, and the “n‐step” method for the enhancement in substrate coverage, bulk recombination could be largely reduced in photoanode, giving excellent PEC water oxidation performances. Figure 8D shows SEM images of one‐step prepared bilayer BVO film and three‐step prepared bilayer BVO film. It indicates that BVO film fabricated through the three‐step method shows largely increased coverage, contributing to improved light absorption. Figure 8E shows that bilayer BVO films exhibit considerably improved *J*–*V* characteristics than single layer BVO films due to the developed twin structure.

Electrodeposited Cu_2_O, ZnO, SnO_2_, CdTe, and Cu_2_ZnSnS_4_ (CZTS) thin films are known to have direct optical bandgap and high absorption coefficient, and these properties make them applicable to various structures of solar cells.^[^ ^144^, ^145^, ^146^, ^147^, ^148^ ^]^ Hsu et al. fabricated homostructural Cu_2_O solar cells through successive electrochemical depositions of a p‐Cu_2_O thin film and n‐Cu_2_O thin film layer on a transparent ITO substrate.^[^ ^149^ ^]^ The n‐type and p‐type Cu_2_O films were grown in acidic and basic electrolytes, respectively, and their electrodeposition conditions were optimized at different coulomb numbers with having suitable bandgap for effective absorption of solar light. Crystalline‐silicon solar cell is also dominantly gaining attention in the photovoltaics market by its advantages of raw‐material abundance, small band gap, and non‐toxicity.^[^ ^148^, ^150^, ^151^ ^]^ To further commercialize silicon solar cells, enhanced solar power conversion efficiency with economical module costs is needed.^[^ ^152^, ^153^, ^154^, ^155^ ^]^ In order to avoid high silicon wafer production costs from the large energy consumption process, direct silicon production from liquid/molten salts at low temperatures has been developed.^[^ ^156^, ^157^, ^158^, ^159^ ^]^ Zou et al. demonstrated direct electrodeposition of superior purity crystalline silicon film from calcium chloride‐based molten salt.^[^ ^150^ ^]^ Unlike traditional silicon electrodeposition from molten fluoride salts, which possesses impurity problems, utilization of calcium chloride allowed the effective synthesis of p‐type, n‐type, and p‐n junction silicon films with tunable thickness for high power conversion efficiency of the solar cell. Figure 8F(i,ii) shows SEM images of p‐type and n‐type silicon films with optimized thickness and morphology through electrodeposition conditions. Figure 8G shows *J*–*V* characteristics of solar cell devices based on the electrodeposited p‐type silicon films. Figure 8H shows the cost advantage of silicon films by summarizing the dependence of total module cost on the module efficiency.

### 4.3 Catalysts

Electrodeposition is also widening its availability into the catalyst synthesis of transition metal, metal oxides, metal hydroxides, and even single‐atom catalysts. These electrodeposited catalysts can be applied to various energy storage and conversion fields.^[^ ^29^, ^160^, ^161^, ^162^, ^163^, ^164^ ^]^ Cost‐effective and earth‐abundant transition metal oxides catalysts like Co_3_O_4_, MnO_2_, NiO are intensively studied to expedite many chemical processes.^[^ ^165^, ^166^, ^167^, ^168^ ^]^ Those metal oxides can be electrochemically optimized on the nanoscale, which can be an effective alternative for high‐price and scarce noble metal catalysts. Sulaiman et al. showed poly(3,4‐ethylenedioxythiophene)/graphene oxide/cobalt oxide nanocomposite based supercapacitor, fabricated by one‐step chronoamperometry electrodeposition method.^[^ ^169^ ^]^ It exhibited excellent capacitance, cyclic stability with largely reduced charge transfer resistance, and superior electronic conductivity. Choi et al. reported electrodeposited Ag‐Cu dendritic structures electrocatalysts, which possess a large surface area for the efficient reduction of CO_2_ into CO.^[^ ^170^ ^]^ They maximized electrochemical properties of Ag‐Cu catalysts by morphological and compositional control in the electrodeposition process, leading to the 2.2 times higher CO_2_ reduction catalytic activity of optimized Ag‐Cu than Ag dendrite catalysts. Vega‐Cartagena et al. demonstrated high‐performance oxygen reduction reaction (ORR) of Ag‐Pd bimetallic nanocatalysts synthesized through rotating disk slurry electrode (RoDSE) electrodeposition method.^[^ ^171^ ^]^ Ag‐Pd catalysts fabrication processes composed of (1) alternated, (2) sequential, and (3) simultaneous Ag and Pd electrodeposition on unsupported Vulcan XC‐72R. They revealed that the 4‐electron pathway proceeds in Ag‐Pd bimetallic, resulting in increased ORR activity than Ag/Vulcan and Pd/Vulcan. Su et al. proposed the effect of various electrodeposition factors on the electrode for nitrogen reduction reaction, aiming to improve nitrogen yield.^[^ ^172^ ^]^ They utilized different electrodeposition programs in terms of the batch of metal solution and charging step, which affected surface morphology and crystallite size. The best nitrogen reduction capability was achieved at the batch solution of SnCl_2_ and PdCl_2_ as the molar ratio of 4 to 1, which exhibited 100% nitrate reduction and 81% nitrogen yield.

**FIGURE 9:**
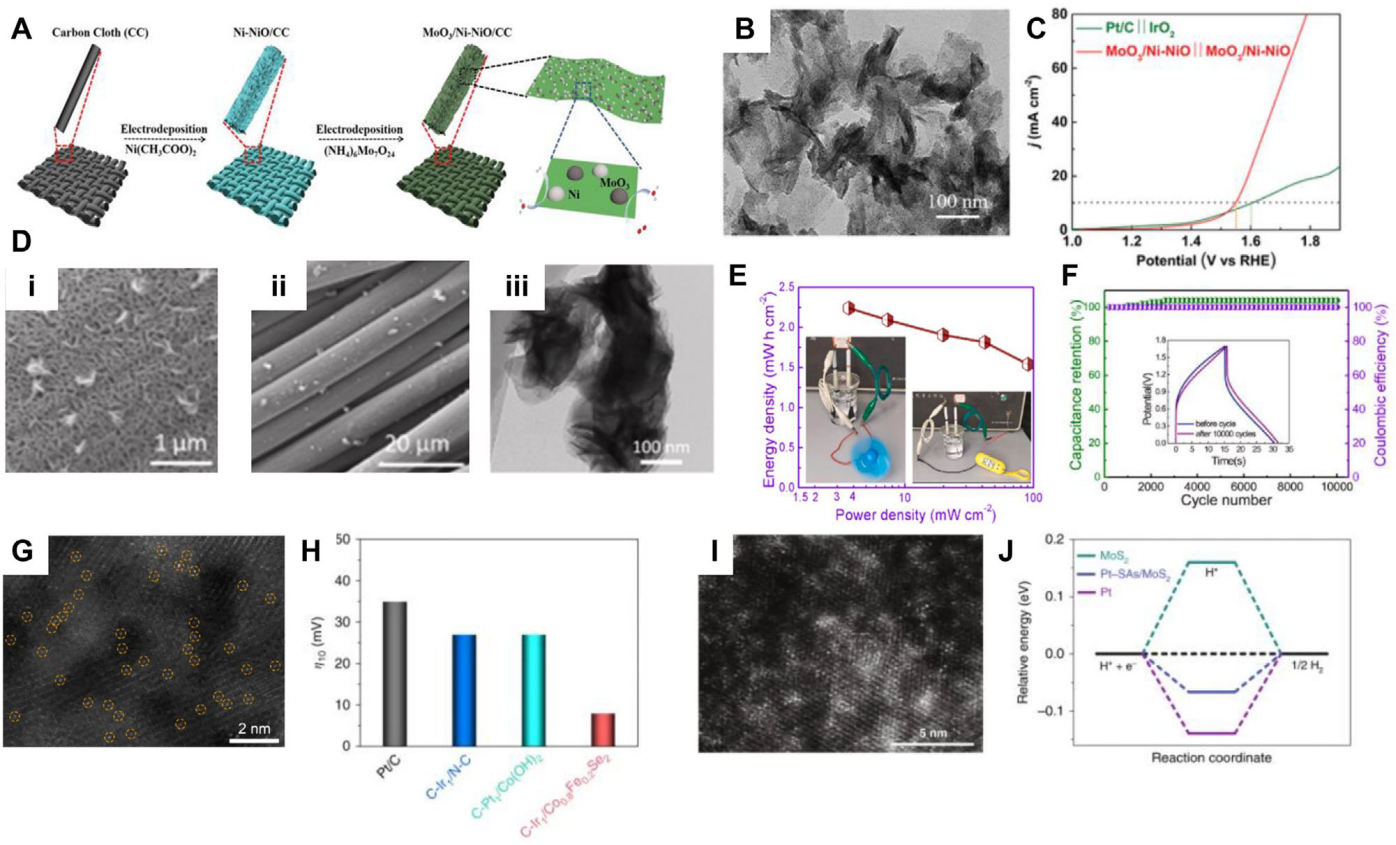
(A) Schematic illustration for the fabrication of MoO_3_/Ni–NiO on carbon cloth. (B) TEM image of MoO_3_/Ni–NiO. (C) Polarization curves for overall water splitting. (A–C) Adapted with permission.^[^ ^173^ ^]^ Copyright 2020, Wiley‐VCH. SEM images of NiCo(NA)‐LDH@ACC (D(i)) at higher and (D(ii)) at lower magnification. (D(iii)) TEM image of NiCo(NA)‐LDH. (E) Ragone plots of the ASC device (inset: turn the small fan for 30 s, make the electronic watch work for 1 h). (F) cyclic stability of the ASC device at 250 mA cm^−2^ for 10,000 cycles (inset: charge‐discharge curves before and after cycles). (D–F) Adapted with permission.^[^ ^177^ ^]^ Copyright 2021, Elsevier. (G) HAADF‐STEM images of C‐Ir_1_/Co(OH)_2_. (H) The overpotentials at 10 mA cm^−2^ for HER. (G,H) Adapted with permission.^[^ ^162^ ^]^ Copyright 2020, Nature Publishing Group. (I) HAADF‐STEM image of Pt‐SAs/MoS_2_ (single Pt atoms highlighted by red circles). (J) Calculated the free energy diagram of the HER at the equilibrium potential for MoS_2_, Pt‐SAs/MoS_2_, and Pt (pH 0). (I,J) Adapted with permission.^[^ ^38^ ^]^ Copyright 2020, Nature Publishing Group

Li et al. proposed a sequentially electrodeposited multi‐component metal oxide structure of MoO_3_/Ni‐NiO where two heterostructures coexist (Ni‐NiO and MoO_3_‐NiO).^[^ ^173^ ^]^ Density functional theory (DFT) calculations verified that Ni‐NiO heterointerfaces are the excellent hydrogen evolution reaction (HER) catalytic active sites, and MoO_3_‐NiO can intensively enhance OER kinetics, showing bifunctional catalytic activity. Not only in the respective OER and HER performances, Ni‐NiO and MoO_3_‐NiO nanohybrid materials cooperatively expedite the overall water splitting process with exhibiting long‐term durability. In combination with experimental results and DFT calculation, outstanding electrocatalytic performance could be clarified as the synergistic effect of abundant heterointerfaces and fully exposed active sites. Figure 9A shows schematics of the MoO_3_/Ni–NiO nanohybrid synthesis process. After the first electrodeposition process, Ni‐NiO composite is deposited on the carbon cloth, and a multi‐component MoO_3_/Ni‐NiO having dual heterointerfaces is fabricated after the second electrodeposition process. Figure 9B shows a TEM image of MoO_3_/Ni‐NiO. It can be seen that numerous MoO_3_ and Ni nanoparticles of 5–10 nm size are dispersed on NiO nanosheets. Figure 9C shows polarization curves of overall water splitting. Overall water splitting electrolyzer with fabricated bifunctional catalyst derived current density of 10 mA cm^–2^ at 1.55 V, superior to that of Pt/C ‖ IrO_2_ electrode (1.60 V).

Transition metallic layered double hydroxides (LDHs) is one of the promising electrochemical catalyst materials by its merits of high conductivity through 2D layered structure, multiple oxidation states, large surface areas, tunable compositions, and high redox activity. LDHs are composed of positive charge brucite‐like layers with divalent and trivalent metal cations and interlayers with charge compensation anions.^[^ ^29^, ^160^, ^161^, ^164^, ^174^ ^]^ Yang et al. synthesized CoFe LDHs coupled with NiFe LDHs nanosheet array supported on nickel foam through facile hydrothermal and electrodeposition method, which showed remarkable performance as water splitting bifunctional electrocatalysts. Its high performance is derived from the enhanced charge transport and mass transport in the 3D interconnected architectures.^[^ ^175^ ^]^ Zhu et al. reported NiCo LDHs electrodeposited on hollow CNTs derived from ZIF‐8 MOF for excellent supercapacitor.^[^ ^176^ ^]^ NiCo LDHs on CNTs formed nanotube arrays. The inner hollow tubular structure constructed effective ion exchange pores and suppressed the impact of volume‐shrinkage during multi‐cycle charging and discharging. Synergistically, the outer NiCo‐LDHs nanosheets provide outstanding capacity ability. Han et al. proposed high mass loading of NiCo‐LDHs nanosheet arrays on activated carbon cloth prepared by electrodeposition for the excellent performance asymmetric supercapacitor.^[^ ^177^ ^]^ For the further enhancement of LDHs in cycle stability and electrochemical performance, methods of growing LDHs on the conductive substrate have been researched in the supercapacitor field.^[^ ^178^, ^179^ ^]^ The three‐dimensional structure substrate itself can allow large specific surface area with rapid transfer channels for electrons and ions, leading to largely improved conductivity. Han and coworkers developed the widely used carbon cloth substrate through a hydrophobic activation process using the nitric oxidation method. Subsequently, NiCo‐LDH was electrodeposited on activated carbon cloth using Ni/Co nitrate and acetate, contributing to cyclic stability improvement. Also, they succeeded in mass loading the electrode through prolongation of deposition time, resulting in superior current density and areal capacitance. Figure 9D shows SEM images of NiCo LDHs nanosheet arrays on activated carbon cloth at higher magnification (Figure 9D(i)) and lower magnification (Figure 9D(ii)) and corresponding TEM image (Figure 9D(iii)). Figure 9E shows the dependence of energy density on power density which is the key parameter for evaluating energy storage devices. This result surpassed recently reported asymmetric supercapacitor assembled devices. Figure 9F shows cyclic stability of fabricated asymmetric supercapacitor device. Even after 10,000 cycles at 250 mA cm^–2^ current density, the synthesized device maintains 104% of the initial capacitance, and the coulombic efficiency remains 100%.

Single‐atom catalysts (SACs) are arising as a hot topic field as a new generation catalyst due to their exceptional catalytic performance ascribed to maximum utilization of reactive sites, specific electronic structure, quantum size effect, distinctive coordinated environment, and strong metal–support interaction. These special properties of SACs are now widely applicated in diverse fields, including CO_2_ hydrogenation, water splitting, and methane conversion.^[^ ^180^, ^181^, ^182^, ^183^, ^184^ ^]^ However, durably anchoring single metals on the substrate is a major issue in fabricating SACs by the surface free energy derived aggregation.^[^ ^185^, ^186^ ^]^ Electrodeposition is emerging as a facile method for effectively depositing SACs on the substrate with high precision. Table 3 shows the comparison of HER performance with electrodeposited SACs. Zhang et al. introduced electrodeposited Au SACs on NiFe LDH substrate and investigated the oxygen evolution reaction activity in SACs.^[^ ^187^ ^]^ Single‐atom Au could be electrochemically deposited on NiFe LDH by applying stepped and stepped back potential. Through DFT calculations, they figured out that Au SACs contributed to lower the energy of rate‐determining step in oxygen evolution reaction by transforming the electronic environment. Zhang et al. reported electrochemical deposition as the general strategy for producing a variety of SACs.^[^ ^162^ ^]^ They achieved electrodeposition of more than 30 different SACs on Co(OH)_2_ and Co_0.8_Fe_0.2_Se_2_ substrates through the cathodic or anodic deposition. Interestingly, the cathodically deposited SACs facilitated hydrogen evolution reaction, while those from anodic deposition promoted oxygen evolution reaction. In addition, the two‐electrode cell consists of cathodically and anodically deposited Ir SACs on Co_0.8_Fe_0.2_Se_2_@Ni foam showed superior overall water splitting performance in the alkaline electrolyte. Figure 9G shows HAADF‐STEM images of cathodically deposited Ir on Co(OH)_2_. It could be revealed that isolated Ir atoms were uniformly dispersed on the substrate. Figure 9H shows an overpotential comparison for HER at 10 mA/cm^2^. Cathodically deposited Ir SACs on substrates revealed higher HER activity than commercial Pt/C. Shi et al. demonstrated site‐specific electrodeposition of atomically dispersed non‐noble and noble metal catalysts onto the TMDs substrate.^[^ ^38^ ^]^ Single‐layer coverage was proceeded by UPD, and it could resist metallic bonding. Furthermore, TMDs have isolated chalcogen sites, stabilizing the metal–support interaction and facilitate the self‐termination growth of single metals during the UPD process. Shi and coworkers also conducted first‐principle calculations to elucidate the catalytic effect of single metals on substrate and change in electronic structure at an atomic level. Figure 9I shows the HADDF‐STEM image of Pt single atoms on MoS_2_. It is shown that Pt single atoms were atomically distributed on MoS_2_ substrate. Figure 9J shows the calculated free energy diagram for hydrogen adsorption in HER at the equilibrium potential for MoS_2_, Pt‐single atoms/MoS_2_, and Pt. Through the immobilization of Pt single atom on MoS_2_, Pt‐single atoms/MoS_2_ can have more favorable HER behavior than commercial Pt catalyst. Favorable HER behavior through the Pt single atom can be attributed to the improvement of the dominance of d‐electron orbitals near the Fermi level. Also, Pt‐SAs/MoS_2_, with a higher Fermi level and lower work function, exhibit the higher electronic energy, enabling the increased electrons to provide capability and better conductivity.

**TABLE 3:** Comparison of HER properties of electrodeposited SACs

**Electrocatalysts**	**Electrolytes**	**Overpotential (mV)(at *j* = 10 mA cm^–2^)**	**Ref**.
C‐Ir_1_/Co_0.8_Fe_0.2_Se_2_	1 M KOH	8	^[^ ^162^ ^]^
Ru@Co‐SAs/N‐doped carbon	1 M KOH	7	^[^ ^193^ ^]^
Ru@carbon quantum dots	1 M KOH	10	^[^ ^194^ ^]^
Co‐substituted Ru	1 M KOH	13	^[^ ^195^ ^]^
Ru@nitrogenated 2D carbon	1 M KOH	17	^[^ ^196^ ^]^
Ru/N‐doped carbon	1 M KOH	21	^[^ ^197^ ^]^
Ru@N‐doped carbon	1 M KOH	26	^[^ ^198^ ^]^
RuCo@N‐doped carbon	1 M KOH	28	^[^ ^199^ ^]^
Ru‐MoO_2_	1 M KOH	29	^[^ ^200^ ^]^
Ru@N‐doped carbon	1 M KOH	32	^[^ ^201^ ^]^
Pt‐SAs/WS_2_	0.5 M H_2_SO_4_	32	^[^ ^38^ ^]^
A‐Ni‐C	0.5 M H_2_SO_4_	34	^[^ ^202^ ^]^
Pt_1_/OLC	0.5 M H_2_SO_4_	38	^[^ ^203^ ^]^
Pt@PCM	0.5 M H_2_SO_4_	105	^[^ ^204^ ^]^
Pt‐GDY2	0.5 M H_2_SO_4_	65	^[^ ^205^ ^]^
Pt_1_@Fe‐N‐C	0.5 M H_2_SO_4_	60	^[^ ^206^ ^]^
Pt/MoS_2_	0.5 M H_2_SO_4_	145	^[^ ^207^ ^]^
Pd/MoS_2_	0.5 M H_2_SO_4_	89	^[^ ^208^ ^]^

## 5 SUMMARY AND PERSPECTIVE

In accordance with the rapidly changing nano‐industry, a technical methodology that can effectively and elaborately cover diverse engineering fields is imperatively needed. Electrodeposition can be a powerful synthesis method for the upcoming nanoscale era for its virtues of wide availability, large‐scale synthesis, material flexibility, ease of control, and cost‐effectiveness. Dimension of structure from 0D to 3D with various phases can be formed through delicate and real‐time experimental controls in the electrodeposition process including applied potential type, deposition overpotential, electrolytes, and additives. Also, 3D conformal deposition on various substrates is possible with the intrinsic electrodeposition property, such as direct‐ion attachment from the electrolyte to the substrate.

Through the versatility of electrodeposition, numerous phases of metal (hydro)oxides, metals, and MOFs can be fabricated. Also, diverse deposition modes such as cyclic voltammetric, pulsed electrodeposition, and UPD with solution modifications can implement various morphologies and even atomic scale deposits. In the light of facile morphology and thickness control, the nanoscale electrodeposition method is widely utilized for high‐performance energy storage and conversion applications like Li batteries, photoelectrochemical water splitting electrodes, solar cells, and electrocatalysts.

**FIGURE 10:**
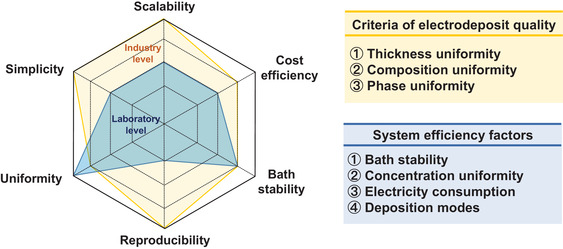
Comparison of nanoscale electrodeposition between laboratory and industrial level

The nanoscale electrodeposition has been actively studied at the laboratory scale as above. The following factors shown in Figure 10 should be considered for the expansion and application to the actual industry. Uniform electrodeposits should be obtained on a large scale (scalability) with price competitiveness, simplicity, and reproducibility. The quality of the electrodeposit can be evaluated in terms of thickness, composition, and phase uniformity. As large‐scale electrodeposition can cause variations in these factors by location, securing scalable conformality of the electrodeposition is one of the challenging issues. It is also required to improve the efficiency of the overall deposition system, including the bath stability, bath concentration uniformity, electricity consumption, and the type of deposition modes. Therefore, it is necessary to improve electrodeposition quality while securing the efficiency of the system. We expect that industrial electrodeposition can enhance productivity and cost‐efficiency rather than existing laboratory‐level electrochemical synthesis techniques.

The electrodeposition technique has not only been reached to the nanoscopic or atom‐scopic region, but also the development of advanced electrodeposition by the combination of other technical fields. As an example, ultrasound‐assisted electrodeposition has the potential to prevent the agglomeration of particles through cavitation and decrease surface roughness.^[^ ^188^ ^]^ Through the grafting of 3D printing technology and electrodeposition, 3D nanostructures from micro to nanoscale have been reported.^[^ ^189^, ^190^ ^]^

Although electrodeposition has been studied for a long time in many scientific areas, we believe this technique retains the limitless potential to further advance in synthesizing materials necessary for future industrial society. More technical investigation and trials in electrodeposition through DFT calculations, enhancing the precision of variable control, and exact understanding of the role of electrodeposited products would be key strategies for surpassing the sphere of application.

## CONFLICT OF INTEREST

Ho Won Jang is a member of the *Exploration* editorial board. The authors declare no conflict of interest.
